# KLF6-mediated recruitment of the p300 complex enhances H3K23su and cooperatively upregulates SEMA3C with FOSL2 to drive 5-FU resistance in colon cancer cells

**DOI:** 10.1038/s12276-025-01424-1

**Published:** 2025-03-13

**Authors:** Bishu Zhang, Tuoya Qi, Jiewei Lin, Shuyu Zhai, Xuelong Wang, Leqi Zhou, Xiaxing Deng

**Affiliations:** 1 Department of General Surgery, Pancreatic Disease Center, Ruijin Hospital, Shanghai Jiao Tong University School of Medicine, Shanghai, China; 2 Research Institute of Pancreatic Diseases, Shanghai Jiao Tong University School of Medicine, Shanghai, China; 3 State Key Laboratory of Oncogenes and Related Genes, Shanghai, China; 4 Institute of Translational Medicine, Shanghai Jiao Tong University, Shanghai, China; 5 Jinshan Hospital of Fudan University, Shanghai, China; 6 Department of Thoracic Surgery, Shanghai Pulmonary Hospital, Tongji University School of Medicine, Shanghai, China; 7 Lingang laboratory, Shanghai, China; 8 Shanghai Changhai Hospital, Naval Medical University, Shanghai, China

**Keywords:** Colon cancer, Computational biology and bioinformatics

## Abstract

Histone lysine succinylation, an emerging epigenetic marker, has been implicated in diverse cellular functions, yet its role in cancer drug resistance is not well understood. Here we investigated the genome-wide alterations in histone 3 lysine 23 succinylation (H3K23su) and its impact on gene expression in 5-fluorouracil (5-FU)-resistant HCT15 colon cancer cells. We utilized CUT&Tag assays to identify differentially enriched regions (DERs) of H3K23su in 5-FU-resistant HCT15 cells via integration with ATAC-seq and RNA sequencing data. The regulatory network involving transcription factors (TFs), notably FOSL2 and KLF6, and their downstream target genes was dissected using motif enrichment analysis and chromatin immunoprecipitation assays. Our results revealed a strong positive correlation between H3K23su DERs, differentially expressed genes (DEGs) and H3K27ac, indicating that H3K23su enrichment is closely related to gene activation. The DEGs associated with the H3K23su GAIN regions were significantly enriched in pathways related to colorectal cancer, including the Wnt, MAPK and p53 signaling pathways. FOSL2 and KLF6 emerged as pivotal TFs potentially modulating DEGs associated with H3K23su DERs and were found to be essential for sustaining 5-FU resistance. Notably, we discovered that FOSL2 and KLF6 recruit the PCAF–p300/CBP complex to synergistically regulate *SEMA3C* expression, which subsequently modulates the canonical Wnt–β-catenin signaling pathway, leading to the upregulation of *MYC* and *FOSL2*. This study demonstrated that H3K23su is a critical epigenetic determinant of 5-FU resistance in colon cancer cells, exerting its effects through the modulation of critical genes and TFs. These findings indicate that interventions aimed at targeting TFs or enzymes involved in H3K23su modification could represent potential therapeutic strategies for treating colorectal cancers that are resistant to 5-FU treatment.

## FOSL2 and KLF6 drive H3K23su-related cancer pathways

Colon cancer is a major health concern worldwide, with treatments such as chemotherapy often facing challenges due to drug resistance. This study explores why colon cancer cells become resistant to the common chemotherapy drug 5-fluorouracil. The researchers focused on a process called lysine succinylation and its role in drug resistance. Using advanced techniques such as CUT&Tag (a method to study protein–DNA interactions), they examined changes in specific proteins in colon cancer cells resistant to 5-fluorouracil. They found that certain proteins, including FOSL2 and KLF6, help to maintain this resistance by affecting gene expression through pathways such as Wnt–β-catenin. The study concludes that targeting these proteins and pathways could help to overcome drug resistance in colon cancer. Future research may focus on developing new treatments that disrupt these processes, potentially improving outcomes for patients with resistant colon cancer.

This summary was initially drafted using artificial intelligence, then revised and fact-checked by the author.

## Introduction

Colon cancer is one of the leading causes of cancer-related morbidity and mortality worldwide^1,2^. Currently, radiotherapy, chemotherapy and surgery are the main strategies for treating recurrent and metastatic colon cancer. Although chemotherapy can relieve symptoms and improve the survival rate of patients, acquired resistance develops in nearly all responsive patients with colon cancer and represents one of the major causes of failure of chemotherapeutic treatment^3^. The development of potential and efficient therapeutic strategies for colon cancer is urgently needed to improve the prognosis of patients.

Although several potential chemotherapeutic and biological agents have been developed to improve the survival and quality of life of patients with colon cancer, 5-fluorouracil (5-FU) remains the recommended first-line therapeutic drug of choice after more than 30 years of clinical research^2,4^. 5-FU is the most widely used chemical for colon cancer therapy and exerts its cytotoxic effects through the inhibition of thymidylate synthase and incorporation of its metabolites into RNA and DNA within cancer cells^5^. However, acquired resistance to 5-FU frequently occurs in patients with colon cancer^6,7^. As such, a comprehensive understanding of the mechanisms underlying 5-FU resistance, coupled with the exploration of safe and effective pharmacological agents capable of reversing this resistance, holds substantial clinical importance and could substantially advance the therapeutic landscape for colorectal cancer.

Despite the wealth of research on genes, noncoding RNAs, histone modifications and signaling pathways associated with 5-FU resistance reported previously in colon cancer^8–10^, the epigenetic regulatory mechanism of lysine succinylation, especially the role of H3K23su, in differential gene expression associated with cancer drug resistance remains poorly understood. As a newly discovered histone posttranslational modification, lysine succinylation is similar to the well-known acetylation modification and appears to accumulate at transcriptional start sites (TSSs) to promote the expression of genes, including H3K79su and H3K122su (refs. ^11–13^). In recent years, the extensive application of cleavage under targets and tagmentation (CUT&Tag) has increased the convenience of detecting genome-wide histone modifications and TF-binding sites (TFBSs) and is increasingly being used for genome-wide profiling of histone modifications owing to its relatively high signal-to-noise ratio, good reproducibility and low input cell number requirements^14,15^.

The present study aimed to decipher the molecular mechanisms underlying 5-FU resistance, a common challenge in colorectal cancer treatment. We identified DERs of H3K23su and investigated their associations with differentially expressed genes (DEGs) in 5-FU-resistant cells using CUT&Tag coupled with assay for transposase-accessible chromatin using sequencing (ATAC-seq) and RNA sequencing (RNA-seq). Our findings indicate a positive correlation between H3K23su differentially enriched regions (DERs) and DEGs, suggesting a potential modulatory role for this histone modification in gene expression patterns related to drug resistance. Furthermore, our analysis of key signaling pathways revealed that several pathways and DEGs are positively related to H3K23su DERs, suggesting a complex interplay between histone modifications and cellular signaling in the context of chemotherapy resistance. We also observed an enrichment of transcription factor (TF) motifs within H3K23su DERs, suggesting that potential regulatory targets are influenced by H3K23su changes. Notably, our results highlight the importance of two TFs, FOSL2 and KLF6, in maintaining resistance to 5-FU. We found that FOSL2 collaborates with KLF6 to regulate the expression of *SEMA3C*, a protein that has been implicated in cancer progression. Moreover, we discovered that FOSL2 upregulates *SEMA3C* and *c-Myc* in 5-FU-resistant HCT15 cells through the canonical Wnt–β-catenin signaling pathway, a critical pathway known to drive cancer growth and survival. Finally, we elucidated the mechanism whereby KLF6 recruits the p300 complex to increase H3K23 succinylation, contributing to the transcriptional alterations associated with 5-FU resistance. These findings underscore the importance of histone succinylation in the epigenetic regulation of the chemotherapy response and provide novel insights into the molecular underpinnings of drug resistance in colorectal cancer.

## Materials and methods

### Cell culture and transfection

Parental (Pa) and 5-FU-resistant (FR) HCT15 cells (HCT15-Pa and HCT15-FR, respectively) were cultured under standard conditions at 37 °C with 5% CO_2_ and 90% humidity as described previously^16^. Both cell lines were cultured in RPMI 1640 medium (Gibco), supplemented with 1% sodium pyruvate (Gibco), 1% penicillin–streptomycin solution (Sangon Biotech) and 10% fetal bovine serum (KEL Biotech). 5-FU was purchased from Sigma-Aldrich, dissolved in dimethyl sulfoxide at a concentration of 20 mg/ml (1000×) and stored at −20 °C. According to the manufacturer’s protocol (iCell Bioscience), HCT15-Pa cells were cultured in drug-free medium, whereas HCT15-FR cells were maintained in 5-FU at a concentration of 20 μg/ml. The cells were seeded in a 10-cm culture dish, expanded to 80–90% confluency and passaged every 5–7 days, with the medium changed every 2–3 days. Before any further experiments were carried out, the HCT15-Pa cells were cultured in parallel with the HCT15-FR cells for comparison, and the HCT15-FR cells were maintained in 5-FU-free medium for 3 days. Lipofectamine 3000 (Invitrogen) was used for transfections. All the small interfering RNA sequences used in this study are listed in Supplementary Table 7.

### CUT&Tag

The CUT&Tag assay was performed as described previously^14,17^ with modifications using the Hyperactive Universal CUT&Tag Assay Kit for Illumina Pro (TD903, Vazyme Biotech) according to the manufacturer’s protocol. In brief, for each capture experiment, approximately 50,000 fresh cells were incubated with 10 μl of prewashed concanavalin-A-coated beads in single low-bind PCR tubes. Fifty microliters of antibody buffer (20 mM HEPES pH 7.5, 150 mM NaCl, 0.5 mM spermidine, 0.05% digitonin, 2 mM EDTA, 0.1% bovine serum albumin and 1× protease inhibitor cocktail) with 1 μg of antibody was added. The samples were further cultured overnight at 2–8 °C, and the beads were then washed twice with 200 μl of Dig-wash buffer (20 mM HEPES pH 7.5, 150 mM NaCl, 0.5 mM spermidine, 0.05% digitonin and 1× protease inhibitor cocktail). Then, 50 μl of Dig-wash buffer containing 1 μg of secondary antibody was added, and the mixture was cultured for 30–60 min at room temperature. After washing three times with Dig-wash buffer, 100 μl of pA/G-Tnp (a final concentration of 0.04 μM) in Dig-300 buffer (20 mM HEPES pH 7.5, 300 mM NaCl, 0.5 mM spermidine, 0.01% digitonin and 1× protease inhibitor cocktail) was added to resuspend the beads. The samples were incubated with continuous rotation for 1 h at room temperature and washed twice with 200 μl of Dig-wash buffer. Then, 50 μl of prepared tagmentation mixture was added, and the samples were incubated for 1 h at 37 °C. A constant amount of spike-in control DNA was added to each library according to the manufacturer’s protocol. The DNA was subsequently extracted and purified with proteinase K and DNA Extract Beads (TD903, Vazyme Biotech) for 30 min at 55 °C. The purified DNA fragments were resuspended in 20 μl of ddH_2_O, after which they could be stored at −20 °C for long periods. For library amplification, a total volume of 50 μl of the sample was placed in a thermocycler using the following program: 72 °C for 3 min; 95 °C for 3 min; 12–15 cycles of 98 °C for 10 s and 60 °C for 5 s; 72 °C for 1 min; and holding at 4 °C. To purify the PCR products, 100 μl of VAHTS DNA Clean Beads (N411, Vazyme Biotech) were added to the above PCR products, which were subsequently incubated at room temperature for 10 min, washed twice with 200 μl of freshly prepared 80% ethanol and eluted in 30 μl of ddH_2_O. Finally, the libraries were sequenced on an Illumina NovaSeq platform (Novogene Biotech), and 150-bp paired-end reads were generated.

For H3K23su or H3K27ac, CUT&Tag was performed as described above using H3K23su (PTM-422, PTM Biolabs) or H3K27ac (ab4729, Abcam) primary antibodies and goat anti-rabbit IgG H&L (Ab206, Vazyme Biotech) as the secondary antibody.

### CUT&Tag data processing

Raw CUT&Tag data were assessed for quality control using FastQC (version 0.11.6). The sequencing read data were aligned to the human reference genome assembly hg19 using Bowtie2 (ref. ^18^). Unmapped reads, secondary alignments and duplicate reads were removed using SAMtools^19^, and only properly paired, uniquely mapped reads retained in the BAM files were used for subsequent data analysis. Picard tools (v.2.9.4) and the ggplot2 R package^20^ were used to calculate and plot the fragment length distributions obtained from paired-end sequencing. Peak calling was performed using MACS2 callpeak (version 2.1.2)^21^. For H3K27ac CUT&Tag data, broad peaks were identified by MACS2 [18] with the --broad parameter. The HOMER v4.10 (ref. ^22^) annotatePeaks script was then used to annotate the location of a given peak in terms of genomic features, such as promoter TSSs, TESs (transcription end sites), introns and exons. For normalization and visualization, the filtered, sorted and scaled BAM files were converted to bigWig format using the bamCoverage script in deepTools (v.2.0)^23^ with --normalizeUsing RPKM -bs 20; then, heatmaps and profile plots were generated using the deepTools computeMatrix, plotHeatmap and plotProfile functions.

### Identification of DERs

To address genome-wide changes in H3K23su and H3K27ac, BEDTools v2.30.0 (ref. ^24^) and deepTools 2.0 (ref. ^23^) were used to compute average normalized RPKM (Reads Per Kilobase of exon model per Million mapped reads) values for CUT&Tag peaks in each group. The genomic regions were defined as GAIN if the peak values (RPKM) showed a >2-fold change in HCT15-FR cells compared with HCT15-Pa cells. The LOSS regions were inversely correlated with decreased CUT&Tag signals in HCT15-FR cells, which presented a fold change greater than 2 in HCT15-Pa cells. For visualization of DERs, ChIPseeker^25^ was used to display genomic loci, and the plotHeatmap and plotProfile functions in deepTools 2.0 (ref. ^23^) were applied to generate profile plots and heatmaps expanded to ±2,000 bp surrounding the DER center.

### Correlation analysis between DERs and DEGs

To annotate genomic locations and assign each DER to the nearest genes (within 100 kb of the TSS), the annotatePeaks script from the HOMER package v4.10 (ref. ^22^) was used, which is based on the GENCODE human release hg19. The nearest genes of DERs subsequently overlapped with the DEGs identified from mRNA-seq data^16^. Fold changes in DERs and their nearest DEGs were used to calculate the Pearson correlation coefficient and *P* values using the Hmisc R package (https://hbiostat.org/R/Hmisc/). The VennDiagram^26^ and ggplot2 R (ref. ^20^) packages were then applied to generate Venn diagrams, box plots or scatter plots.

### KEGG pathway enrichment analysis and visualization

Enrichment analysis of the Kyoto Encyclopedia of Genes and Genomes (KEGG) pathway was performed using the clusterProfiler R package^27^ with the hypergeometric distribution test. KEGG pathways with *P* values < 0.05 were considered significantly enriched. Cytoscape version 3.9.0 (http://www.cytoscape.org/) was used to display links between significantly enriched KEGG pathways and their related DEGs.

### TF-binding motif enrichment and occurrence analysis

BEDTools v2.30.0 (ref. ^24^) was used to obtain the CUT&Tag peak summits located in the GAIN or LOSS regions, and then the findMotifsGenome.pl program of the HOMER package v4.10 (ref. ^22^) was used to find known motifs and their corresponding TFBSs surrounding the peak summits with the parameter -size −200,200. HOMER software (v4.10) was also utilized to compute the *P* values for motif enrichment, with a threshold of *P* < 0.01 being designated as statistically significant by default. An integrative analysis of CUT&Tag and RNA-seq datasets was conducted to identify TFs with potential binding sites within H3K23ac GAIN regions, correlating these with upregulated DEGs. Similarly, TFs with potential binding sites within H3K23ac LOSS regions were correlated with downregulated DEGs. TFs exhibiting notable differences in expression levels between HCT15-FR and HCT15-Pa cells were defined as differentially expressed TFs (DETFs). The annotatePeaks.pl function in HOMER v4.10 (ref. ^22^) was then used to calculate the occurrence probability of a certain DETF within a 1-kb region flanking peak summits (from −500 to +500 bp) and to identify potential target DEGs of DETFs (TFBSs located within 2 kb of the TSS), and the outputs were visualized using the ggplot2 R package^20^.

### PPI network and coexpression analysis of DETFs

To obtain information regarding the predicted and experimental interactions of DETFs associated with H3K23su changes, a protein–protein interaction (PPI) network was constructed in Search Tool for the Retrieval of Interacting Genes (STRING) (https://string-db.org/)^28^. Using the Markov cluster algorithm with the default inflation parameter 3.0, we obtained the network and subnetwork modules. Based on gene expression patterns and protein coregulation derived from ProteomeHD^29^, the coexpression scores between DETFs were calculated.

### Cell proliferation assay

The half-maximal inhibitory concentration (IC_50_) values were assessed in CCK-8 assays (Beyotime). HCT15-FR cells with *FOSL2* knockdown, *KLF6* knockdown, and *FOSL2* and *KLF6* knockdown as well as HCT15-Pa cells with *FOSL2* overexpression, *KLF6* overexpression, and *FOSL2* and *KLF6* overexpression were collected after 48 h, and 1.5 × 10^3^ cells were seeded in 96-well plates and incubated further. Then, 90 µl of complete medium supplemented with 10 µl of CCK-8 reagent was added after 5-FU treatment, the mixture was incubated for 2 h and the absorbance was subsequently measured at 450 nm. The inhibition rate (%) was calculated as (*A*_treated_ − *A*_blank_)/(*A*_control_ − *A*_blank_) × 100%, and A represents the number of colonies. For the colony formation assay, 1.5 × 10^3^ HCT15-FR cells with *FOSL2* knockdown, *KLF6* knockdown, and *FOSL2* and *KLF6* knockdown as well as HCT15-Pa cells with *FOSL2* overexpression, *KLF6* overexpression, and *FOSL2* and *KLF6* overexpression were seeded in six-well plates and treated with 5-FU at 10 μM. After 14 days, 1% crystal violet solution was added to fix the cells, and the number of colonies was counted. For the 5-ethynyl-2′-deoxyuridine (EdU) assay, an EdU labeling kit (Epizyme) was used to examine the proliferative ability of the cells. HCT15-FR cells with *FOSL2* knockdown, *KLF6* knockdown, and *FOSL2* and *KLF6* knockdown as well as HCT15-Pa cells with *FOSL2* overexpression, *KLF6* overexpression, and *FOSL2* and *KLF6* overexpression (3 × 10^4^) were seeded in 12-well plates for 48 h. Then, the cells were incubated with EdU reagent for 2 h, fixed with 4% paraformaldehyde and 0.5% Triton X-100, and subjected to Hoechst staining. The EdU incorporation rate was defined as the proportion of EdU-positive cells (red) to total Hoechst 33342-positive cells (blue).

### Western blotting, immunofluorescence and immunoprecipitation

Cell proteins were extracted with RIPA buffer, loaded, separated on 10% or 12.5% SDS–PAGE gels, transferred onto polyvinylidene fluoride membranes and incubated with the indicated primary antibodies (Supplementary Table 8). Gapdh and α-tubulin were used as controls. ECL (enhanced chemiluminescence) reagents were used to examine protein expression. Western blotting reagents were purchased from EpiZyme. Immunofluorescence assays were used to confirm the subcellular localization of FOSL2 and KLF6, KLF6 and p300/CBP-associated factor (PCAF), and KLF6 and p300 in HCT15-FR cells, as previously described^30^. PPIs were confirmed by co-immunoprecipitation (co-IP), as previously described^30^.

### ChIP

The SimpleChIP Plus Sonication Chromatin IP Kit (CST) was used to perform the chromatin immunoprecipitation (ChIP) assay according to the manufacturer’s protocol. The cells were crosslinked with 1% formaldehyde for 10 min at room temperature to ensure that the proteins were crosslinked with DNA. DNA fragments were sheared by sonication. The nuclear lysate was immunoprecipitated with anti-FOSL2, anti-KLF6, anti-H3K23su and anti-Pol II or IgG antibodies. The purified DNA fragments were analyzed by qRT–PCR. The ChIP–qPCR primers used are listed in Supplementary Table 9.

### Flow cytometric analysis

HCT1 cells were collected and incubated with fluorescein-isothiocyanate-conjugated annexin V and propidium iodide for 30 min in the dark at room temperature and were analyzed by flow cytometry, as previously described^31^.

### Mouse model assay

Stably transfected *FOSL2*-knockdown, *KLF6*-knockdown and *FOSL2*- and *KLF6*-knockdown cells were injected subcutaneously into mice (5 × 10^6^ cells per site). The longest longitudinal diameter (*a*) and longest transverse diameter (*b*) were measured every 3 days. The tumor volume was calculated as follows: volume = 1/2(*a* × *b*^2^). The mice were killed after 33 days, and the tumors were weighed.

### Public datasets

ATAC-seq and mRNA-seq data of HCT15-Pa and HCT15-FR cells, including the DEGs and bigWig files used in this study, were obtained from our previous publication and are available in the NCBI Gene Expression Omnibus (GEO) repository under the accession number GSE190951 (https://www.ncbi.nlm.nih.gov/geo/query/acc.cgi?acc=GSE190951) (ref. ^16^).

### Statistical analysis and data visualization

In this study, all differential analyses were conducted with HCT5-Pa cells serving as the control group and HCT5-FR cells as the experimental group. All the statistical analyses and plot generation were performed in R (https://www.R-project.org/) unless otherwise specified. Significance was tested between samples using the Wilcoxon signed-rank test or Student’s *t*-test. *P* values, false discovery rates and fold changes were calculated in the analysis. For correlation analyses, Pearson correlation tests were performed. Differences between experimental groups were considered significant when *P < 0.05, **P < 0.01, ***P < 0.001 and ****P < 0.0001. The representative sequencing tracks of CUT&Tag, ATAC-seq and mRNA-seq were extracted and visualized using the WashU Epigenome Browser (http://epigenomegateway.wustl.edu) (hg19 assembly).

## Results

### Genome-wide changes in H3K23su were positively correlated with differential gene expression in FR colon cancer cells

**Fig. 1:**
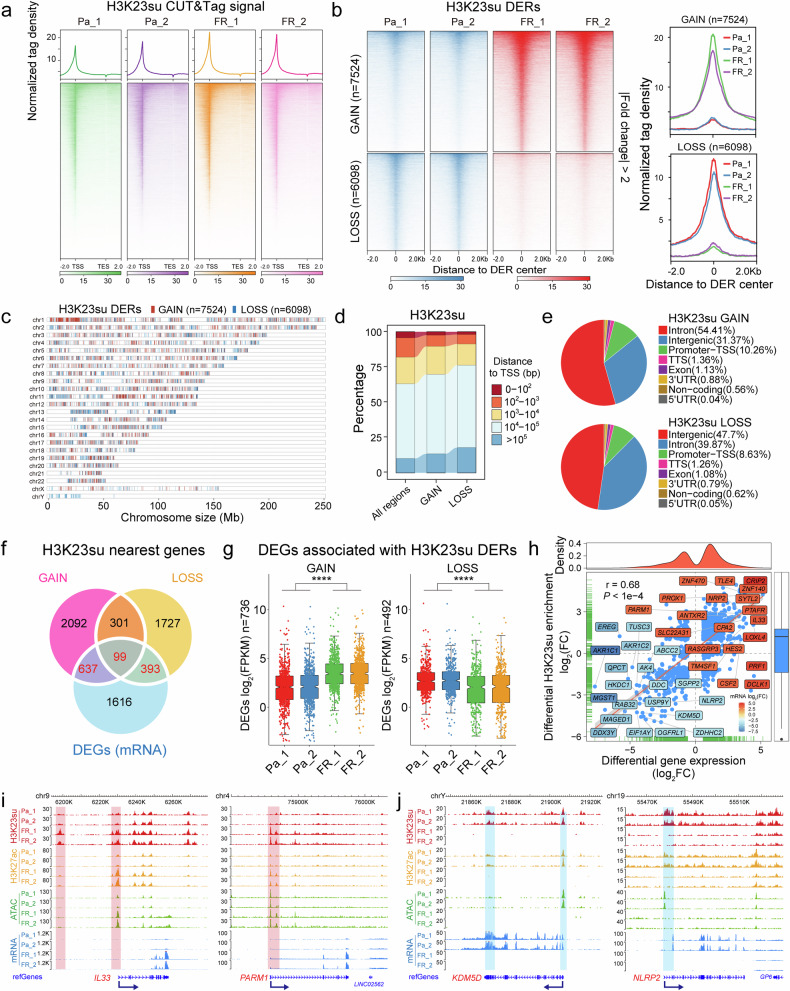
Differential enrichment of H3K23su was positively correlated with differential gene expression in HCT15-FR cells. **a** Average profiles (top) and density heatmaps (bottom) indicating H3K23su CUT&Tag signals across a genomic window 2 kb upstream of the TSS and 2 kb downstream of the TES. **b** Left: heatmap representation of greater (GAIN) and lower (LOSS) H3K23 with CUT&Tag DERs in HCT15-FR cells versus Pa cells. The top panel shows the genomic distribution of 7524 H3K23su GAIN regions; the bottom panel shows the distribution of 6098 H3K23su LOSS regions. The signals are displayed in descending order within the 2-kb window from the center of each DER. Right: average tag density profiles of H3K23su enrichment plotted onto 2-kb regions surrounding the center of DERs. **c** The distribution of GAIN (red) and LOSS (blue) regions for H3K23su enrichment across chromosomes. **d** The distance to the closest gene TSS of all regions and the new GAIN and LOSS regions for H3K23su enrichment. **e** Proportions of GAIN (top) and LOSS (bottom) regions enriched in H3K23su at different genomic loci, such as introns, exons, promoter TSSs, TESs and intergenic regions. **f** Venn diagram illustrating the overlapping genes between the nearest genes of H3K23su DERs (GAIN and LOSS) revealed by CUT&Tag and the DEGs identified by mRNA-seq. **g** Box plots showing the relative mRNA expression levels of DEGs associated with H3K23 in the GAIN (left) and LOSS (right) regions. The notches of boxes represent the medians, and the *P* values were calculated using the Wilcoxon signed-rank test; ****P < 0.0001. **h** Pearson correlation analysis between H3K23su DERs and their nearest DEGs identified in **a**. Each blue dot represents a DEG related to differential H3K23su enrichment. The top- and bottom-ranked 20 DEGs with the most substantial difference based on the log_2_fold change (FC) are shown in different colors. The Pearson correlation coefficient value (*r*) and *P* value are shown in the figure. **i**, WashU Epigenome Browser tracks showing H3K23su (red), H3K27ac (orange), ATAC-seq (green) and mRNA-seq (blue) signals of representative upregulated *IL33* and *PARM1* genes in both the HCT15-Pa and HCT15-FR cell lines, each with two replicates. The H3K23su GAIN regions are shaded in red. The dark-blue arrows indicate the TSS and the direction of gene transcription. **j** Genomic snapshots of H3K23su (red), H3K27ac (orange), ATAC-seq (green) and mRNA-seq (blue) signals of representative downregulated *KDM5D* and *NLRP2* genes. These data are presented for both the HCT15-Pa and HCT15-FR cell lines, with each cell line featuring two replicates. The H3K23su LOSS regions are shaded in blue. The dark-blue arrows indicate the TSS and the direction of gene transcription.

To elucidate the epigenetic modifications of H3K23su in the context of 5-FU resistance in colorectal cancer and to decipher its regulatory role in gene expression, we conducted CUT&Tag assays to profile H3K23su in both the HCT15-Pa and HCT15-FR cell lines (Fig. 1a and Supplementary Table 1). The H3K23su-enriched chromatin was fragmented into patterns characteristic of mono-, di- and trinucleosomes (Supplementary Fig. 1a). The consistency in fragment sizes across samples suggests that chromatin is equally accessible to antibody-directed transposases, and more than 60,000 H3K23su peaks were identified for each sample (Supplementary Table 2). Further functional genomic annotation analysis revealed that more than 90% of the H3K23su peaks were enriched in the promoter TSSs, introns and intergenic regions (Supplementary Fig. 1b). Moreover, H3K23su was markedly enriched within the −2 kb to +2 kb region surrounding the gene TSSs (Fig. 1a), implying a potential association between H3K23su and the activation of target gene transcription.

Compared with HCT15-Pa cells, we identified 13,622 H3K23su DERs in HCT15-FR cells. Among these regions, 7524 regions presented a more than twofold increase in the H3K23su signal (RPKM), whereas 6098 regions presented a more than twofold decrease in H3K23su; thus, we defined these DERs as GAIN and LOSS (Fig. 1b, c and Supplementary Table 3). The distribution of H3K23su DERs was relatively consistent across particular chromatin regions in HCT15-FR cells (Fig. 1c). Further genomic annotation of H3K23su DERs indicated that largely the distal intergenic or intragenic (intron) regions presented differential H3K23su signals, with a relatively high proportion located in distal regions more than 1,000 bp away from a gene TSS (Fig. 1d, e and Supplementary Table 3), suggesting that H3K23su DERs presumably function through distal regulatory elements such as enhancers.

To assess the impact of H3K23su changes on differential gene expression, we examined the correlation between differential gene expression and differential H3K23su enrichment on the basis of RNA-seq and CUT&Tag data^16^. H3K23su DERs were assigned to nearby genes on the basis of their coordinates in the human genome, and a total of 736 and 492 of the nearest DEGs were found to be associated with the H3K23su GAIN and LOSS regions, respectively (Fig. 1f). Notably, most of the DEGs related to H3K23su GAIN regions were notably upregulated, whereas the DEGs associated with H3K23su LOSS regions presented notably downregulated mRNA levels in all the biological replicates (Fig. 1g), indicating that genes within increased H3K23su signals tended to have increased transcription levels. Moreover, analysis of the Pearson correlation coefficient confirmed that the changes in gene expression were positively (*r* = 0.68) correlated with H3K23su DERs located within 100 kb of the TSS (Fig. 1h).

**Fig. 2:**
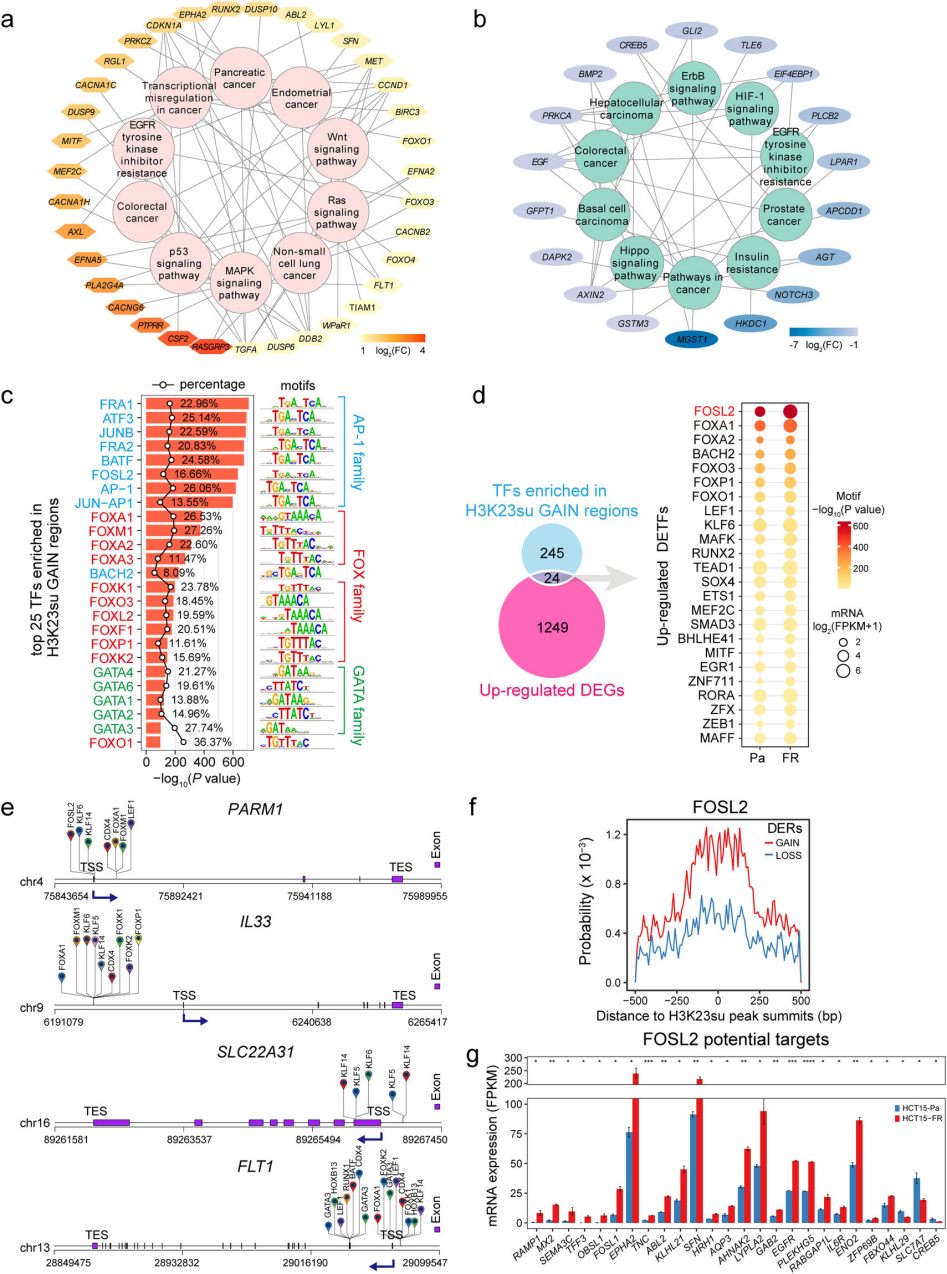
Identification of signaling pathways, TFs and their target genes associated with H3K23su DEGs. **a**, **b** Top 10 KEGG pathway-associated DEGs related to the H3K23su GAIN (**a**) and LOSS (**b**) regions. DEGs are shown in a color gradient based on their log_2_fold change (FC) values. **c** The top 25 enriched known TF motifs of the H3K23su GAIN regions, with *P* values estimated from HOMER (v.4.10). The AP-1, FOX and GATA family members are colored blue, red and green, respectively. The percentages of target sequences of DERs with TF motifs are indicated by polygonal chains in black. **d** Venn diagram showing the overlap between the corresponding TFs for each motif identified by HOMER (v.4.10) in the H3K23su GAIN regions and the upregulated DEGs identified by RNA-seq. The dot plot (right) shows the identified upregulated TFs. The color of each dot represents the *P* value of TF enrichment, and only TFs with *P* values < 0.01 were included. The size of each dot represents the expression level of the corresponding TF, log_2_(FPKM + 1). FPKM, Fragments Per Kilobase of exon model per Million mapped fragments. **e** Predicted TFBSs by HOMER analysis of representative DEGs positively correlated with H3K23su changes. The dark-blue arrows indicate the TSS and the direction of gene transcription. **f**, **g** Top: line charts showing the distribution probability of representative FOSL2-binding motifs around CUT&Tag peak summits in H3K23su DERs. Bottom: histograms indicating the expression levels of predicted TF (FOSL2) target DEGs in H3K23su DERs identified by HOMER (v.4.10). Only DEGs with TFBSs in the promoter-TSS regions were included. DEGs are listed in descending order based on the log_2_fold change.

A previous study revealed that lysine succinylation extensively overlaps with acetylation in prokaryotes and eukaryotes^32^. Here, we also performed CUT&Tag for H3K27ac in both HCT15-Pa and HCT15-FR cells (Supplementary Fig. 1c), which is a robust indicator of enhancer activity for transcriptional activation and is well recognized as a marker for superenhancers^33,34^. Similar criteria were then used to identify H3K27ac DERs, and 8,143 GAIN and 7,636 LOSS regions were obtained from H3K27ac CUT&Tag data (Supplementary Fig. 1d). As expected, the H3K27ac DERs also presented a high correlation coefficient (*r* = 0.73) with their nearest DEGs (Supplementary Fig. 1e, f). Interestingly, we also observed a positive correlation between the trends of gene expression changes in H3K23su and H3K27ac (Supplementary Figs. 1g and 2 and Supplementary Table 3). Combined with previously published ATAC-seq and RNA-seq data^16^, the alterations in H3K23su enrichment, especially in the region near the promoter TSS, were closely related to the differential expression of critical genes involved in 5-FU resistance, including the upregulated *IL33*, *SLC22A31* and *PARM1* genes with increased H3K23su and H3K27ac and chromatin accessibility (Fig. 1i and Supplementary Fig. 3) and the downregulated *KDM5D*, *DDX3Y* and *NLRP2* genes with decreased H3K23su and H3K27ac levels and a hypoaccessible chromatin state (Fig. 1j and Supplementary Fig. 4). These results suggested that alterations in H3K23su enrichment were accompanied by differential gene expression, H3K27ac intensity and chromatin accessibility in FR colon cancer cells. To identify critical signaling pathways associated with H3K23su DERs in HCT15-FR cells, previously identified DEGs correlated with H3K23su GAIN and LOSS regions with an absolute value of log_2_fold change >1 were used for KEGG pathway enrichment analysis. In total, we obtained 53 key DEGs enriched in the top 10 KEGG pathways, including 35 upregulated and 18 downregulated DEGs positively associated with H3K23su DERs (Fig. 2a, b and Supplementary Fig. 5a). The upregulated DEGs associated with the H3K23su GAIN regions, including *RASGRP3*, *MET* and *FOXO1*, were markedly enriched in colorectal cancer, pancreatic cancer and the Wnt, MAPK and p53 signaling pathways (Fig. 2a, Supplementary Fig. 5b and Supplementary Table 4), whereas the H3K23su LOSS regions associated with downregulated DEGs, such as *NOTCH3*, *MGST1* and *GFPT1*, were involved mainly in insulin resistance, colorectal cancer and the HIF-1 and ErbB signaling pathways (Fig. 2b, Supplementary Fig. 5c and Supplementary Table 4). These findings were similar to those concerning the signaling pathways enriched with DEGs associated with H3K27ac DERs (Supplementary Fig. 6). Integration of the H3K27ac CUT&Tag and ATAC-seq datasets revealed that pervasive alterations in H3K23su could modulate the differential gene expression of many components of signaling cascades with concomitant changes in chromatin accessibility and H3K27ac intensity.

### Genome-scale identification of TFs potentially regulating DEGs associated with H3K23su DERs

TFs are known to regulate gene expression through binding to specific motifs in the promoters or enhancers of target genes, which play critical roles in development and disease^35–37^. To identify potential TFs located within H3K23su DERs, we performed known motif enrichment analysis to scan TF motif occurrences and TFBSs^22^. By default, 277 and 157 potential TFs were identified (*P* < 0.01) within the H3K23su GAIN and LOSS regions, respectively (Supplementary Table 5). The results revealed that activator protein-1 (AP-1) family members, including JUN, FOS, MAF and ATF subfamily members, were notably enriched in both the H3K23su GAIN and LOSS regions (Fig. 2c and Supplementary Fig. 7b), which preferentially bind to the DNA sequence 5′-TGACTCA-3′ and play critical roles in various cellular activities and cancer development^36,38,39^. Moreover, the H3K23su GAIN regions were also notably enriched with FOX and GATA family members (Fig. 2c), whereas the H3K23su LOSS regions were enriched with motifs of TEAD family members (Supplementary Fig. 7b). To identify potential regulators of certain DEGs associated with 5-FU resistance, we analyzed the TFBSs for the top 50 TFs potentially enriched in H3K23su DERs. The upregulated DEGs associated with H3K23 in GAIN regions, such as *IL33*, *LOXL4* and *FLT1*, were potentially regulated by FOX, GATA and KLF family members (Fig. 2c, e and Supplementary Fig. 7a). Moreover, the downregulated DEGs related to H3K23su LOSS regions, including *NOTCH3*, *KDM5D* and *MGST1*, might be closely related to the loss of TEAD and KLF family member binding (Supplementary Fig. 7b). These results indicate that the presence or absence of critical TF binding is closely related to the gain or loss of H3K23su and differential gene expression.

To further identify DETFs associated with differential H3K23su intensity and their potential target DEGs, the identified motif cognate TFs with *P* values <0.01 were intersected with the DEGs from the RNA-seq data. Ultimately, a total of 24 upregulated TFs, including FOSL2, FOXA1, RUNX2 and MITF, were found to be associated with H3K23 in GAIN regions (Fig. 2d), whereas H3K23 and LOSS regions were enriched in motifs of 11 downregulated TFs, including HNF1B, KLF3 and GRHL2 (Supplementary Fig. 7c). Analysis of the occurrence probability for a given motif confirmed that FOSL2 and FOXA1 presented a greater binding probability around the CUT&Tag peaks within H3K23su GAIN regions (Fig. 2f and Supplementary Fig. 7a), whereas the motifs of HNF1B and KLF3 were generally distributed more frequently in H3K23su LOSS regions (Supplementary Fig. 7d, e). To identify the target DEGs that are potentially regulated by these DETFs, we obtained the corresponding DEGs of their TFBSs within 2-kb regions surrounding the TSS. The majority of the predicted target DEGs of FOSL2 and FOXA1, such as *RAMP1*, *SEMA3C*, *EGFR* and *FOXP1*, were markedly upregulated in HCT15-FR cells (Fig. 2f, g and Supplementary Figs. 7a and 8), whereas most HNF1B and KLF3 targets, including *AGT*, *AK4*, *CD44* and *DDX3Y*, were downregulated (Supplementary Figs. 7d, e and 8). These results revealed that DETFs and their potential target DEGs associated with H3K23su are altered in HCT15-FR colon cancer cells.

### FOSL2 and KLF6 are essential for maintaining resistance to 5-FU

**Fig. 3:**
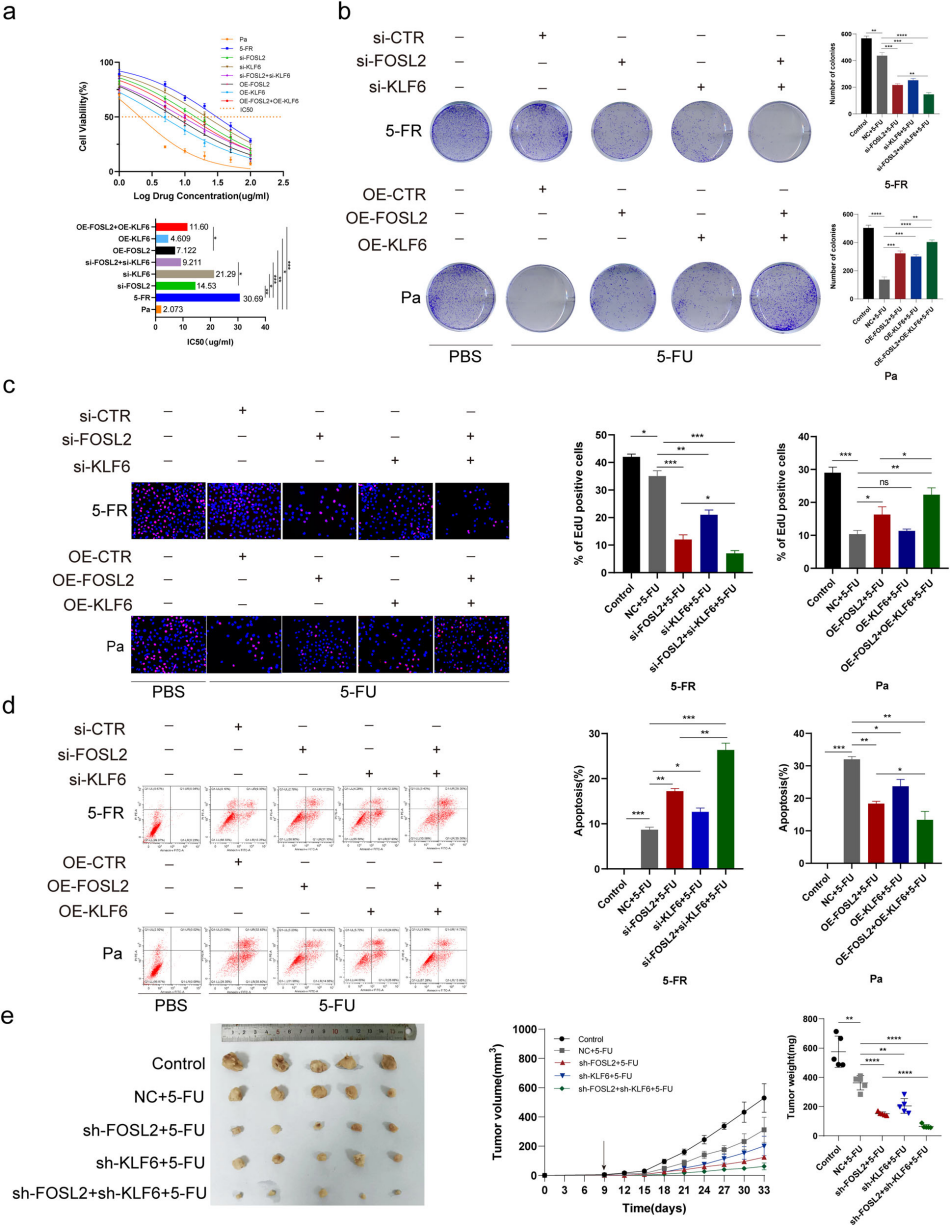
KLF6 cooperated with FOSL2 to maintain the 5-FR resistance of HCT15-FR cells. **a** The IC_50_ values of FOSL2, KLF6, FOSL2 and KLF6 in HCT15-FR cells, *FOSL2*- and *KLF6*-knockdown HCT15-FR cells, and *FOSL2*- and *KLF6*-overexpressing HCT15-Pa cells were calculated after 5-FU administration. These findings demonstrated that *FOSL2* and *KLF6* are essential for maintaining 5-FU resistance in a synergistic manner. **b** HCT15-FR cells, HCT15-FR cells with *FOSL2* knockdown, *KLF6* knockdown, *FOSL2* and *KLF6* knockdown, HCT15-Pa cells, and HCT15-Pa cells with *FOSL2* overexpression, *KLF6* overexpression, and *FOSL2* and *KLF6* overexpression were cultured in six-well plates with 5-FU administration. The results showed that *FOSL2* cooperated with *KLF6* to increase the viability of HCT15-FR cells. **c** An EdU assay was used to examine the viability of HCT15-FR cells and HCT15-Pa cells after transfection and treatment with 5-FU. These results indicated that *FOSL2* cooperated with *KLF6* to increase the percentage of EdU-positive HCT15-FR cells. **d** A flow cytometry apoptosis assay was performed to examine the apoptosis rates of HCT15-FR cells and HCT15-Pa cells after transfection and treatment with 5-FU. HCT15-Pa and HCT15-FR cells were incubated with fluorescein-isothiocyanate-conjugated annexin V and propidium iodide followed by incubation in the dark at room temperature for 30 min. The results revealed that *FOSL2* cooperated with *KLF6* to decrease the apoptosis rates of HCT15-FR cells. **e** Subcutaneous tumors formed from HCT15-FR cells, HCT15-FR cells with *FOSL2* knockdown, *KLF6* knockdown, and *FOSL2* and *KLF6* knockdown. The tumor volume was measured every 3 days after injection. The tumor weights of the subcutaneous xenografts are shown. These results suggest that *FOSL2* cooperated with *KLF6* to maintain 5-FU resistance in a synergistic manner.

We further sought to understand the molecular mechanisms of certain critical DETFs, FOSL2 and KLF6, in FR colon cancer cells. The IC_50_ values were determined in GraphPad software after the administration of 5-FU (Fig. 3a). The IC_50_ value in HCT15-FR cells was greater than that in HCT15-FR cells after the knockdown of *FOSL2* and *KLF6* and the combined knockdown of *FOSL2* and *KLF6* (Fig. 3a). Notably, the IC_50_ value in HCT15-FR cells with *FOSL2* knockdown exceeded that in HCT15-FR cells with simultaneous *FOSL2* and *KLF6* knockdown (Fig. 3a). By contrast, the IC_50_ value in HCT15-Pa cells was lower than that in HCT15-Pa cells with *FOSL2* overexpression, *KLF6* overexpression and concomitant overexpression of *FOSL2* and *KLF6* (Fig. 3a). Furthermore, the IC_50_ value in HCT15-Pa cells with *FOSL2* overexpression was considerably lower than that in HCT15-FR cells with *FOSL2* and *KLF6* overexpression (*P* < 0.05; Fig. 3a). To evaluate the viability of HCT15-FR cells, HCT15-Pa cells and transfected cells after treatment with 5-FU, we implemented colony formation assays in conjunction with EdU incorporation. The results indicated that the viability of HCT15-FR cells was notably enhanced after the administration of 5-FU. By contrast, HCT15-FR cells with *FOSL2* knockdown, *KLF6* knockdown and combined knockdown of *FOSL2* and *KLF6* presented a diminished capacity for survival under the same conditions. Conversely, the viability of HCT15-Pa cells overexpressing *FOSL2*, *KLF6* or both was greater than that of unmodified HCT15-Pa cells. In addition, simultaneous knockdown of *FOSL2* and *KLF6* in HCT15-FR cells resulted in a lower viability rate than that observed in cells with *FOSL2* knockdown alone. Furthermore, the overexpression of both *FOSL2* and *KLF6* in HCT15-Pa cells increased the viability rate after 5-FU treatment compared with that in cells overexpressing only *FOSL2* (*P* < 0.05; Fig. 3b, c). Subsequently, HCT15-Pa and HCT15-FR cells were incubated with fluorescein-isothiocyanate-conjugated annexin V and propidium iodide, incubated in the dark at room temperature for 30 min, and analyzed by flow cytometry. The knockdown of *FOSL2* and *KLF6* and the combined knockdown of *FOSL2* and *KLF6* resulted in a substantial increase in apoptotic events in HCT15-FR cells after 5-FU administration. Conversely, the overexpression of *FOSL2*, *KLF6* or both markedly decreased the rate of apoptosis in HCT15-Pa cells compared with that in their nonoverexpressing counterparts after treatment with 5-FU. Moreover, the apoptotic response in HCT15-FR cells with *FOSL2* knockdown alone was less pronounced than that in cells in which both *FOSL2* and *KLF6* were knocked down. Similarly, the overexpression of *FOSL2* in HCT15-Pa cells led to a decrease in apoptosis, which was further mitigated by the additional overexpression of *KLF6* (*P* < 0.05; Fig. 3d). These results indicated that the targeted knockdown of *FOSL2* and *KLF6* markedly decreased the resistance of HCT15-FR cells to 5-FU. Furthermore, our data revealed a collaborative interaction between *KLF6* and *FOSL2* in the maintenance of 5-FU resistance in HCT15-FR cells. In accordance with these findings, individual and combined knockdown of *FOSL2* and *KLF6* compromised the 5-FU resistance of HCT15-FR cells in a nude mouse subcutaneous tumor model. Notably, the dual knockdown of *FOSL2* and *KLF6* had a more pronounced effect on diminishing 5-FU resistance than did the single knockdown of *FOSL2*. (*P* < 0.05; Fig. 3e). In addition, by IHC, we confirmed that the expression levels of Ki-67 and cyclin D1 (CCND1) were considerably decreased as the drug resistance of colon cancer cells decreased (Supplementary Fig. 9a).

### FOSL2 cooperated with KLF6 to regulate SEMA3C expression

**Fig. 4:**
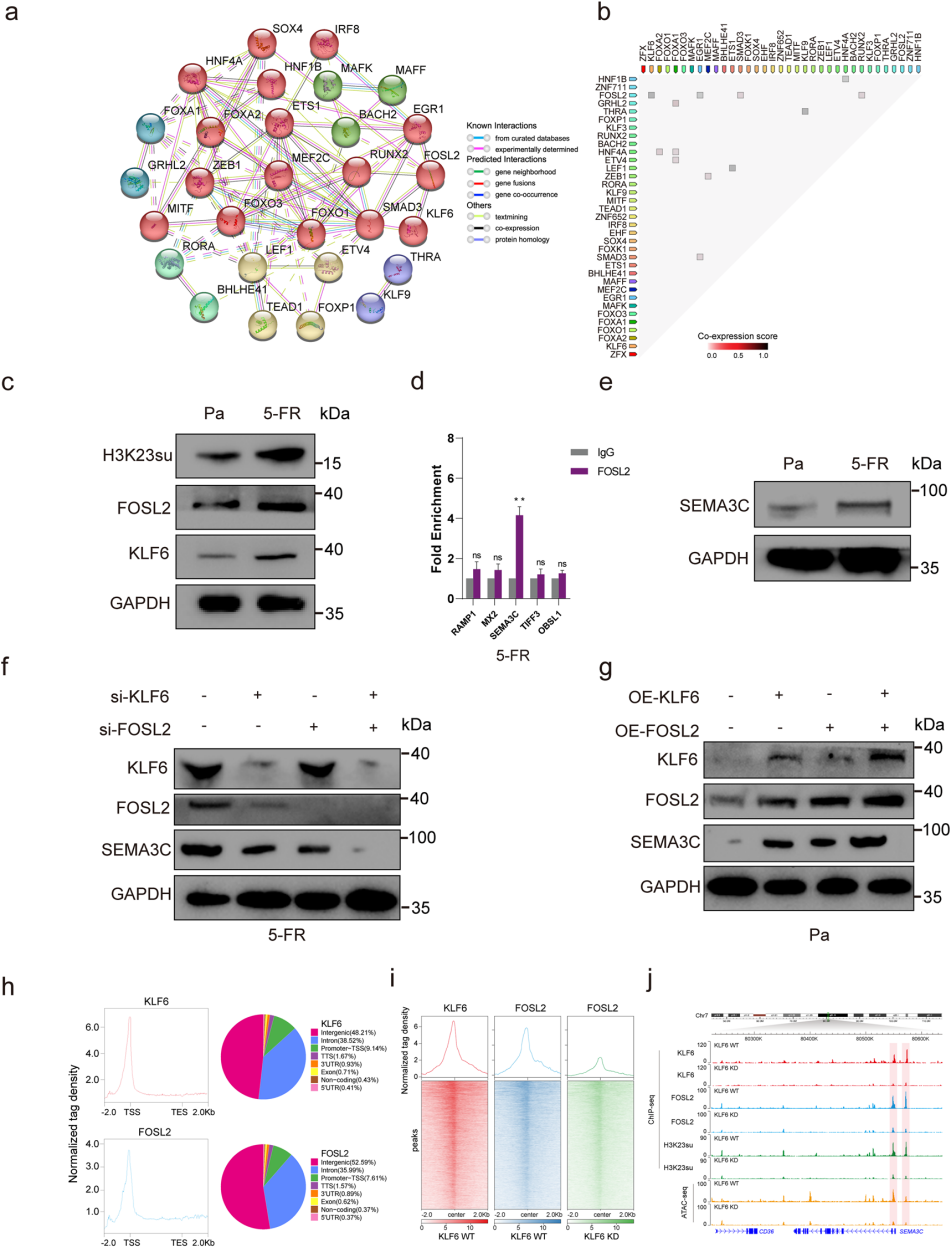
KLF6 cooperated with FOSL2 to regulate SEMA3C expression. **a** PPI network illustrating DETFs associated with H3K23su DERs. The network nodes indicate proteins, and the colored edges represent evidence of different types of PPIs. Strong relationships and networks are indicated by solid lines and were clustered using the Markov algorithm with the default settings. **b** Coexpression of DETFs associated with H3K23su DERs in *Homo sapiens* identified using STRING. **c** H3K23su, FOSL2 and KLF6 expression levels in HCT15-FR cells were greater than those in HCT15-Pa cells, as determined by western blotting. **d** ChIP–qPCR confirmed that the *SEMA3C* promoter was enriched in the FOSL2 binding site. **e** SEMA3C expression was greater in HCT15-FR cells than in HCT15-Pa cells. **f** Expression levels of KLF6, FOSL2 and SEMA3C in the NC (negative control), *FOSL2*-knockdown, *KLF6*-knockdown, and *FOSL2*- and *KLF6*-knockdown groups of HCT15-FR cells. SEMA3C expression decreased with FOSL2 or KLF6 knockdown. The decrease in *SEMA3C* expression was more marked when *FOSL2* and *KLF6* were knocked down together. **g** Protein levels of FOSL2, KLF6 and SEMA3C in the NC, *FOSL2*-overexpression, *KLF6*-overexpression, and *FOSL2*- and *KLF6*-overexpression groups of HCT15-Pa cells. *SEMA3C* expression increased as *FOSL2* or *KLF6* overexpression increased, and the increase in *SEMA3C* expression was more marked when *FOSL2* and *KLF6* were overexpressed together in HCT15-Pa cells. **h** FOSL2 and KLF6 were highly enriched near the TSS. **i** Intensive colocalization relationship between *KLF6* and *FOSL2*. **j** *KLF6* knockout impaired FOSL2 and H3K23su binding to the promoter of *SEMA3C*.

To elucidate the PPI network and coexpression profiles of the DETFs, the STRING database was utilized to dissect both established and predicted direct physical interactions, as well as indirect functional associations among proteins^28^. The PPI network revealed that the clustered and upregulated FOSL2/SMAD3/EGR1, ETS1/LEF1 and ZEB1/MEF2C proteins potentially cooperate to regulate the expression of DEGs associated with H3K23 in GAIN regions in HCT15-FR cells. Conversely, the diminished binding affinity of HNF1B/HNF4A and KLF9/THRA was implicated in the downregulation of key DEGs (Fig. 4a, b). Moreover, relatively high coexpression scores were also observed for FOSL2/KLF6, FOSL2/RUNX2 and GRHL2/FOXA1 (Fig. 4b), suggesting that the DETFs related to H3K23su DERs might work together and be responsible for 5-FU resistance in HCT15 colon cancer cells.

We detected that the protein levels of H3K23su, FOSL2 and KLF6 in HCT15-FR cells were greater than those in HCT15-Pa cells by western blot (Fig. 4c and Supplementary Fig. 14a). Notably, compared with the differential protein levels of FOXA1, FOXA2 and BACH2, that of FOSL2 was more pronounced in HCT15-FR cells, as shown in Fig. 4c and Supplementary Figs. 9a and 14a, which aligns with the bioinformatics findings shown in Fig. 2d. ChIP–qPCR was then used to examine the FOSL2 binding site. We confirmed that the *SEMA3C* promoter enrichment factor was notably higher in HCT15-FR cells than in control cells (*P* < 0.05; Fig. 4d). We subsequently detected that the expression of *SEMA3C* was greater in HCT15-FR cells than in HCT15-Pa cells (Fig. 4e and Supplementary Fig. 14b). We then examined the expression of *SEMA3C* in HCT15-FR cells with *FOSL2* knockdown, *KLF6* knockdown, and *FOSL2* and *KLF6* knockdown. The results indicated that *SEMA3C* expression decreased as *FOSL2* or *KLF6* was knocked down. In addition, *SEMA3C* decreased more obviously when *FOSL2* and *KLF6* were knocked down together. Similarly, SEMA3C increased as *FOSL2* or *KLF6* overexpression increased, and *SEMA3C* increased more obviously as *FOSL2* and *KLF6* were overexpressed together in HCT15-Pa cells. Notably, *FOSL2* decreased with *KLF6* knockdown, whereas *FOSL2* increased with *KLF6* overexpression (Fig. 4f, g and Supplementary Fig. 14c, d). We conducted ChIP–seq and found that FOSL2 and KLF6 were highly enriched near the TSS (Fig. 4h). The ChIP–seq peaks of KLF6 and FOSL2 were distributed at the promoter TSSs by 9.14% and 7.61%, respectively. In addition, gene profile analysis revealed an intense colocalization relationship between KLF6 and FOSL2 proteins (Fig. 4i). Compared with wild-type *KLF6* cells, *KLF6* knockout impaired FOSL2 and H3K23su binding to the promoter of *SEMA3C* (Fig. 4j).

### FOSL2 upregulated SEMA3C and c-Myc in HCT15-FR cells via the canonical Wnt–β-catenin signaling pathway

**Fig. 5:**
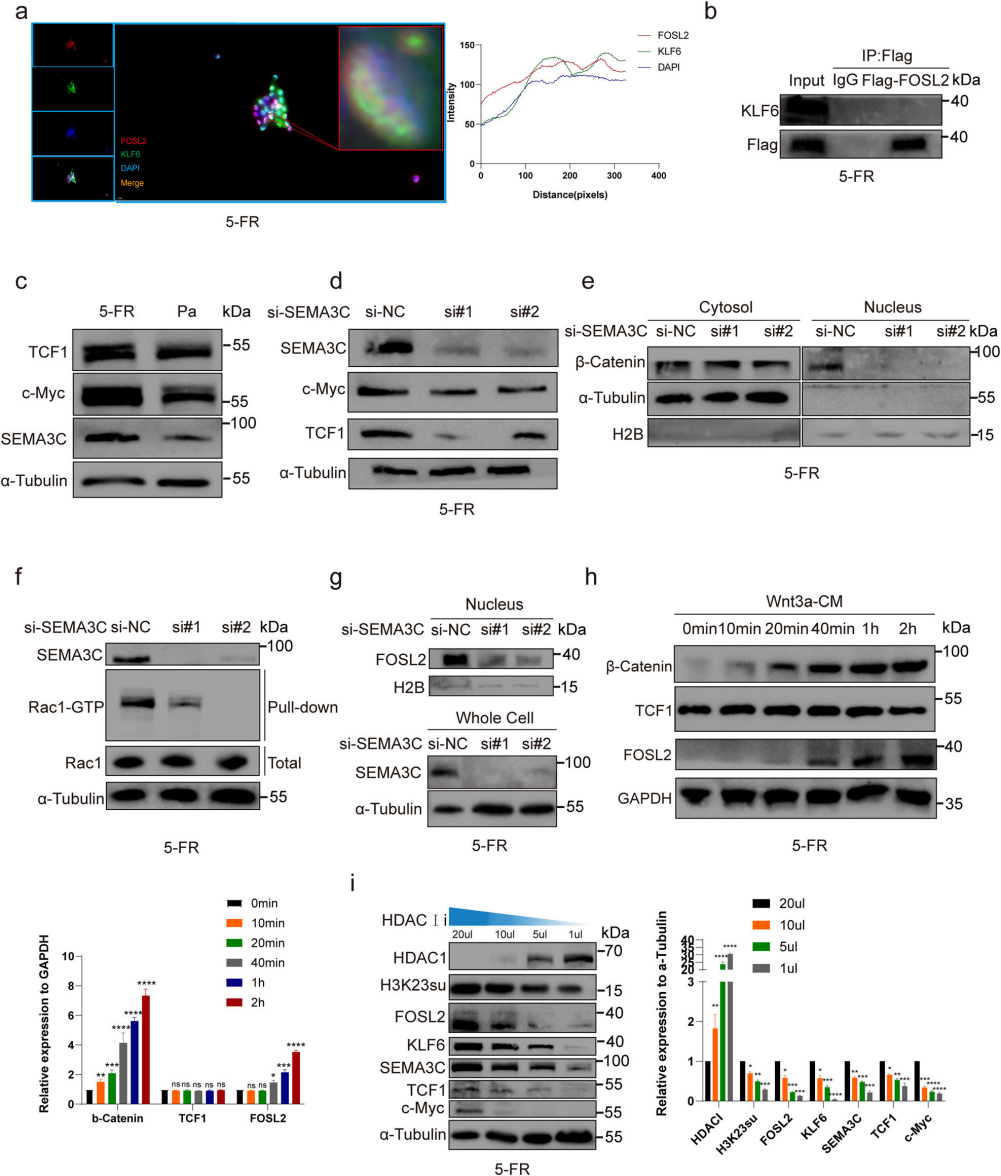
FOSL2 upregulated SEMA3C and c-Myc in HCT15-FR cells via the canonical Wnt–β-catenin signaling pathway. **a** Colocalization of FOSL2 and KLF6 in the nucleus of HCT15-FR cells by immunofluorescence. **b** Co-IP revealed no interaction between FOSL2 and KLF6. **c** TCF1 and c-Myc were upregulated in HCT15-FR cells, as shown by western blotting. **d** TCF1 and c-Myc decreased as *SEMA3C* was knocked down in HCT15-FR cells, as shown by western blotting. **e** β-Catenin markedly decreased in the nucleus following *SEMA3C* knockdown in HCT15-FR cells, as shown by western blotting. **f** Rac1–GTP was reduced in HCT15-FR cells when *SEMA3C* was knocked down, as shown by a Rac1–GTP pulldown assay. **g** FOSL2 expression was reduced in the nucleus in HCT15-FR cells with *SEMA3C* knockdown. **h** FOSL2 was upregulated as Wnt–β-catenin was activated by Wnt3a-CM. TCF1 was not influenced by β-catenin. **i** H3K23su, FOSL2, KLF6, SEMA3C, TCF1 and c-Myc expression levels increased as HDAC was suppressed.

Fluorescence confocal microscopy revealed the colocalization of FOSL2 and KLF6 in the nuclei of HCT15-FR cells (Fig. 5a). We provide images with larger magnification and their details in Supplementary Fig. 11a. HCT15-FR cells were transfected with Flag-FOSL2, and the proteins were immunoprecipitated with a Flag monoclonal antibody. However, a co-IP assay indicated that FOSL2 could not be precipitated with KLF6 (Fig. 5b). Previous studies have shown that *SEMA3C* can activate the canonical Wnt pathway^40^. Numerous studies have shown that the Wnt–β-catenin signaling pathway is abnormally activated in various tumors and participates in tumor occurrence and progression by regulating downstream target genes such as *c-Myc* and *cyclin D1* (refs. ^41–43^). Sustained activation of the Wnt signaling pathway is the fundamental cause of many colon cancers. The activation of this pathway leads to the accumulation of β-catenin in the nucleus and the formation of complexes with lymphocyte enhancer factor 1 (LEF1) and TFs of the T cell factor (TCF) family, thereby activating the transcription of target genes such as *MYC* and *CCND1* (ref. ^44^). Previous studies have shown that nitazoxanide can reverse drug resistance and inhibit the proliferation of colorectal cancer cells by downregulating the Wnt–β-catenin signaling pathway and reducing the expression of *MYC* and *CCND1*. These findings indicate that the Wnt-β-catenin signaling pathway plays an important role in tumor development and drug resistance development^45^. Western blotting was then used to examine the expression of key target Wnt genes, and the results revealed that TCF1 and c-Myc were upregulated in HCT15-FR cells (Fig. 5c and Supplementary Fig. 14e). We subsequently detected that TCF1 and c-Myc decreased as SEMA3C was knocked down (Fig. 5d and Supplementary Fig. 14f). In addition, the level of β-catenin obviously decreased in the nucleus after *SEMA3C* knockdown (Fig. 5e and Supplementary Fig. 14g). A Rac1-GTP pulldown assay was used to confirm that Rac1-GTP was reduced in HCT15-FR cells after *SEMA3C* knockdown (Fig. 5f). Notably, we detected that the FOSL2 level tended to be reduced in the nucleus of HCT15-FR cells with *SEMA3C* knockdown (Fig. 5g and Supplementary Fig. 14h). Previous studies have shown that *FOSL2* is a target gene in the Wnt-β-catenin signaling pathway^46,47^. We hypothesized that *SEMA3C*, which is the target gene of *FOSL2*, could increase the expression level of *FOSL2* via the Wnt–β-catenin signaling pathway. Subsequently, HCT15-FR cells were treated with Wnt3a-conditioned medium (Wnt3a-CM) to activate the Wnt-β-catenin signaling pathway. We then detected that FOSL2 was upregulated by β-catenin, as Wnt–β-catenin was activated by Wnt3a-CM. However, TCF1 was not influenced by β-catenin (Fig. 5h). We speculated that, as previously reported, *FOSL2* might be regulated by β-catenin via TCF-independent transcriptional regulation of Wnt-β-catenin target genes^48^, which was distinguished from the way that *FOSL2* regulates *SEMA3C* and *c-Myc* in HCT15-FR cells via the canonical Wnt–β-catenin signaling pathway. The HCT15-FR cells were then treated with trichostatin A (TSA), an HDAC class I/II inhibitor, at 20, 10, 5 or 1 µM. A recent study showed that the inhibition of HDAC1/2/3 could result in elevated levels of H3K23su^49^. Similarly, H3K23su, FOSL2, KLF6, SEMA3C, TCF1 and c-Myc increased as HDAC1 was suppressed (Fig. 5i).

### KLF6 recruited the p300 complex to enhance the succinylation of H3K23

**Fig. 6:**
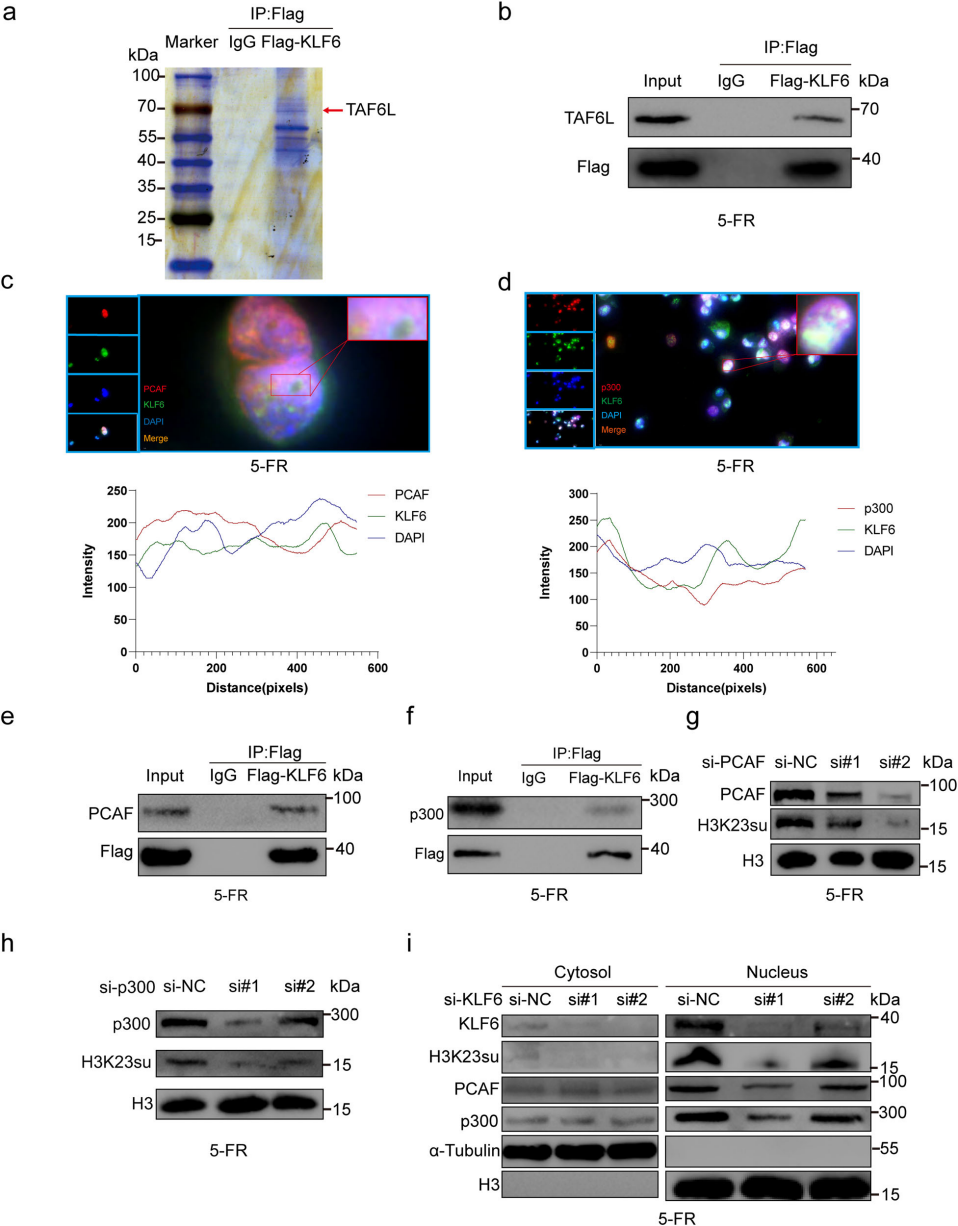
KLF6 recruited the p300 complex to enhance the succinylation of H3K23. **a** IP-MS analysis revealed that TAF6L was at the top of the list. **b** KLF6 interacted with TAF6L, as shown by co-IP analysis. **c** Colocalization of KLF6 and PCAF in the nucleus of HCT15-FR cells by IF. **d** Colocalization of KLF6 and p300 in the nucleus of HCT15-FR cells by IF. **e** KLF6 interacted with PCAF, as shown by co-IP analysis. **f** KLF6 interacted with p300, as shown by co-IP analysis. **g** Western blotting revealed that H3K23su decreased as PCAF was knocked down. **h** Western blotting revealed that H3K23su decreased as p300 was knocked down. **i** H3K23su, PCAF and p300 decreased in the nucleus of HCT15-FR cells with KLF6 knockdown, whereas PCAF and p300 remained unchanged in the cytosol.

Immunoprecipitation (IP) followed by mass spectrometry (MS) analysis revealed that TAF6L, a component of the PCAF histone acetylase complex, was at the top of the list (Fig. 6a and Supplementary Table 6). Co-IP was then performed to confirm that KLF6 interacted with TAF6L (Fig. 6b). PCAF, a histone acetyltransferase (HAT), has binding activity with CBP and p300. In addition, p300/CBP has been verified to succinylate histones^13^. Fluorescence confocal microscopy was performed to detect the colocalization of KLF6 with PCAF or KLF6 with p300 in the nucleus of HCT15-FR cells (Fig. 6c, d). We provide images with larger magnification and their details in Supplementary Figs. 12a and 13a. Co-IP assays suggested that KLF6 interacted with PCAF and p300 (Fig. 6e, f). HCT15-FR cells were then transfected with si-PCAF, si-p300 and si-KLF6 (si, small interfering RNA). We detected that H3K23su decreased as PCAF and p300 were knocked down (Fig. 6g, h and Supplementary Fig. 14i-j). In addition, H3K23su, p300 and PCAF decreased in the nucleus of HCT15-FR cells with KLF6 knockdown but remained unchanged in the cytosol (Fig. 6i and Supplementary Fig. 14k). These findings suggest that p300 and PCAF are recruited to the nucleus by KLF6. CBP and p300 are considered to function identically. They share a high degree of sequence identity in structured domains, including their enzymatic HAT domains and their bromodomains^50^. Owing to their high sequence homology, these two proteins are commonly referred to as CBP/p300, and their activity is often considered largely redundant^51^. This redundant effect always plays major roles in cells, such as in cell protection and apoptosis^51,52^. Moreover, CBP/p300 double knockdown also causes the upregulation of HDAC1 (refs. ^51,53^). Therefore, especially with respect to H3K23su, it is indeed necessary to explore whether p300/CBP plays an important redundant role in enhancing tumor cell survival rather than ignoring the role of CBP because of the structural and functional similarities between p300 and CBP. Consequently, we treated HCT15-FR cells with si-CBP and si-p300. By western blotting, we detected that H3K23su decreased as p300 was knocked down, whereas H3K23su was not affected as CBP was knocked down (Supplementary Fig. 9e). These findings suggest that the H3K23su level is regulated only by p300, which could be considered a preference of p300 for H3K23su in HCT15-FR cells. However, compared with p300 or CBP knockdown, HDAC1 expression was markedly decreased when p300 and CBP were knocked down simultaneously (Supplementary Fig. 9e). These findings indicate that HDAC1 is regulated by the redundant effects of p300 and CBP. Interestingly, we found that both HDAC2 and HDAC3 were not affected by p300 or CBP, even when both p300 and CBP were knocked down (Supplementary Fig. 9e). A previous study reported that the inhibition of HDAC1/2/3 resulted in elevated levels of H3K23su, but inhibition of only HDAC1 did not affect H3K23su^49^. These findings suggest that this redundant roles of p300 and CBP limit the regulation of HDACs in HCT15-FR cells. There are differences in substrate specificity between p300 and GCN5 (ref. ^54^). Although most studies have focused on exploring their respective functions and mechanisms, p300 and GCN5 can each play roles in the same gene regulatory network. Therefore, it is necessary to explore whether *GCN5* affects H3K23su. We treated HCT15-FR cells with si-KAT2A (GCN5). We subsequently found that there was no interaction between KLF6 and KAT2A and that *KAT2A* knockdown did not affect H3K23su levels (Supplementary Fig. 9f, g).

### The decrease in 5-FU resistance induced by FOSL2 knockdown and the increase in 5-FU resistance caused by KLF6 overexpression could be reversed through KLF6 overexpression and p300 knockdown

**Fig. 7:**
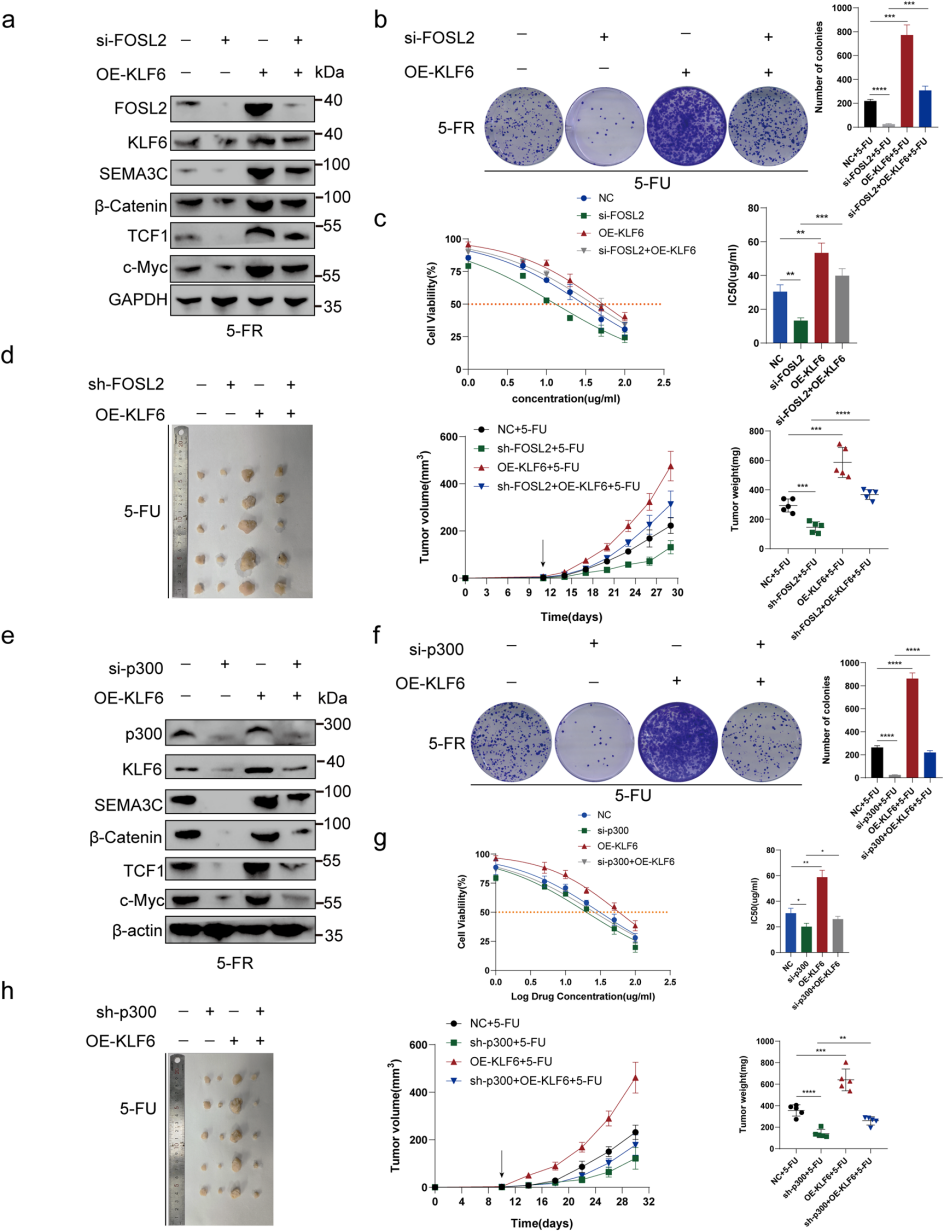
FOSL2 overexpression or knockdown induced an increase or decrease in 5-FU resistance, which could be reversed by KLF6 knockdown or overexpression. **a** *FOSL2* knockdown in HCT15-FR cells downregulated the 5-FU-resistance-promoting proteins SEMA3C, β-catenin, TCF1 and c-Myc. When KLF6 was overexpressed, this downregulation was impaired. **b** The decrease in 5-FU resistance induced by *FOSL2* knockdown in HCT15-FR cells could be rescued by *KLF6* overexpression through colony formation assays. **c** The IC_50_ values suggested that *KLF6* overexpression could reverse the *FOSL2* knockdown-induced decrease in 5-FU resistance in HCT15-FR cells. **d** A nude mouse subcutaneous tumor model indicated that *KLF6* overexpression could rescue the decrease in 5-FU resistance induced by *FOSL2* knockdown. The tumor volume was measured every 3 days after injection. The tumor weights of the subcutaneous xenografts are shown. **e** SEMA3C, β-catenin, TCF1 and c-Myc, which induce an increase in 5-FU resistance in HCT15-FR cells, were downregulated by si-p300. This downregulation was reversed by *KLF6* overexpression. **f** Colony formation assays indicated that *KLF6* overexpression reversed the *p300* knockdown-induced decrease in 5-FU resistance in HCT15-FR cells. **g** The IC_50_ values suggested that *KLF6* overexpression reversed the *p300* knockdown-induced decrease in 5-FU resistance in HCT15-FR cells. **h** A nude mouse subcutaneous tumor model indicated that *KLF6* overexpression rescued the decrease in 5-FU resistance induced by *p300* knockdown. The tumor volume was measured every 4 days after injection. The tumor weights of the subcutaneous xenografts are shown.

*FOSL2* knockdown in HCT15-FR cells downregulated the 5-FU-resistance-promoting proteins SEMA3C, β-catenin, TCF1 and c-Myc. When *KLF6* was overexpressed, this downregulation was impaired (Fig. 7a and Supplementary Fig. 15a). Next, we found that the decrease in 5-FU resistance induced by *FOSL2* knockdown in HCT15-FR cells could be reversed by *KLF6* overexpression through colony formation assays and IC_50_ values (Fig. 7b, c). Moreover, an identical rescue effect was observed in the nude mouse subcutaneous tumor model. The tumor volume was measured every 3 days after injection (Fig. 7d). To examine the rescue effect between KLF6 and p300, we knocked down *EP300* in HCT15-FR cells. SEMA3C, β-catenin, TCF1 and c-Myc, which led to an increase in 5-FU resistance in HCT15-FR cells, were downregulated. This downregulation was impaired by *KLF6* overexpression (Fig. 7e and Supplementary Fig. 15b). Colony formation assays then revealed that the decrease in viability of HCT15-FR cells induced by p300 knockdown could be rescued by *KLF6* overexpression (Fig. 7f). Moreover, the effect of p300 activity was examined by C646–p300 inhibitor and OE-KLF6 (OE, overexpression). By western blotting, we found that C646 decreased 5-FU-resistance-promoting proteins KLF6, SEMA3C, β-catenin, TCF1 and c-Myc, and this decrease was reversed by *KLF6* overexpression (Supplementary Fig. 10a). The colony formation assays suggested that *KLF6* overexpression could reverse the C646-induced decrease in 5-FU resistance in HCT15-FR cells (Supplementary Fig. 10b). Moreover, the nude mouse subcutaneous tumor model indicated that *KLF6* overexpression could rescue the decrease in 5-FU resistance in HCT15-FR cells induced by C646. The tumor volume was measured every 4 days after injection (Supplementary Fig. 10c). The IC_50_ value in HCT15-FR cells with p300 knockdown was lower than that in HCT15-FR cells, which was reversed by *KLF6* overexpression (Fig. 7g). This rescue effect was also observed in a nude mouse subcutaneous tumor model. The tumor volume was measured every 4 days after injection (Fig. 7h). Based on these findings, we investigated the collaboration between KLF6 and FOSL2 via the recruitment of p300 to increase 5-FR resistance in HCT15-FR cells.

### FOSL2 or KLF6 knockdown induced a decrease in 5-FU resistance, which could be rescued by SEMA3C overexpression

**Fig. 8:**
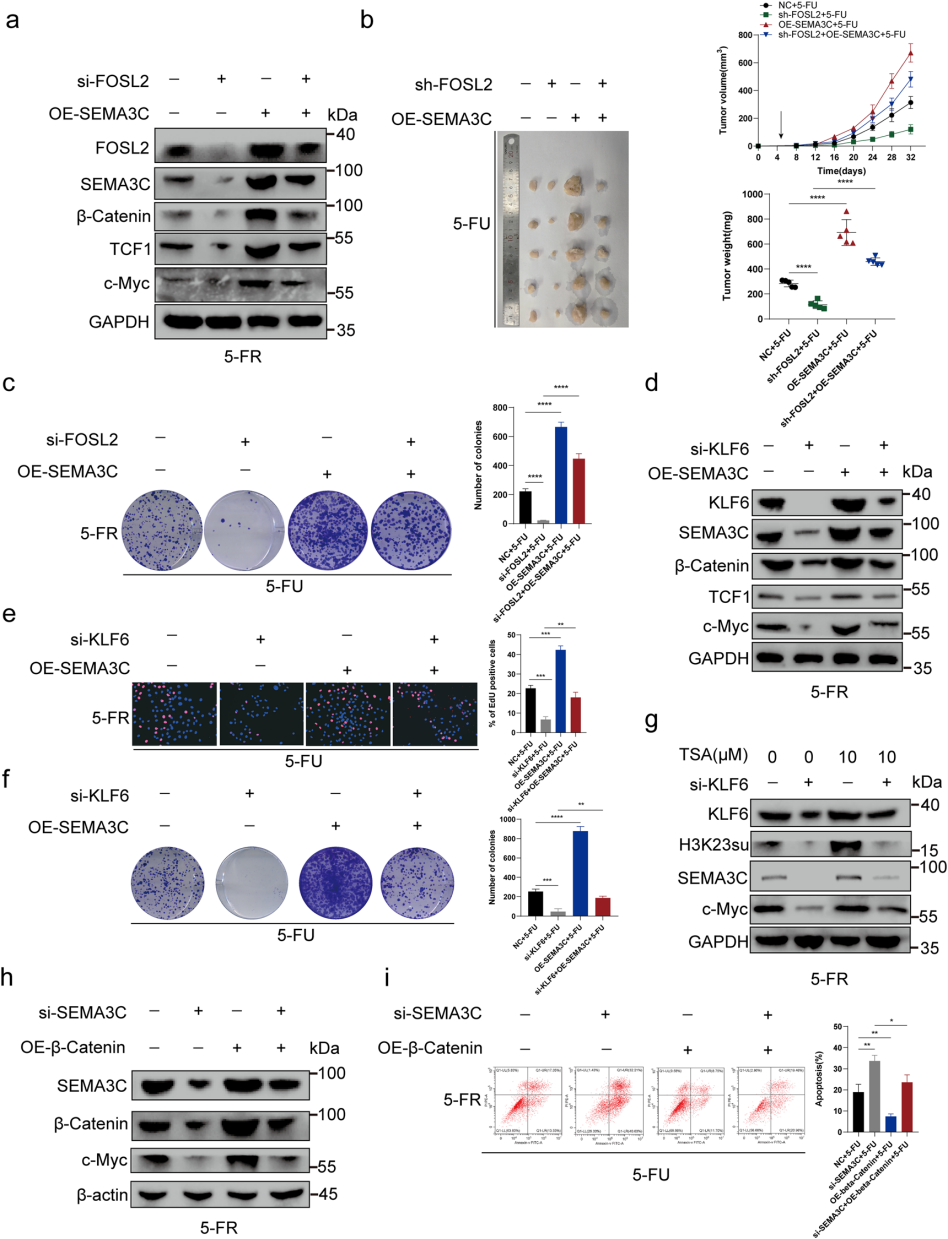
FOSL2 or KLF6 knockdown induced a decrease in 5-FU resistance, which could be rescued by SEMA3C overexpression. **a** *FOSL2* knockdown downregulated the 5-FU-resistance-promoting proteins SEMA3C, β-catenin, TCF1 and c-Myc. When SEMA3C was overexpressed, this downregulation was impaired. **b** The decrease in 5-FU resistance induced by *FOSL2* knockdown in a nude mouse subcutaneous tumor model could be rescued by *SEMA3C* overexpression. The tumor volume was measured every 4 days after injection. The tumor weights of the subcutaneous xenografts are shown. **c** Colony formation assays indicated that *SEMA3C* overexpression reversed the *FOSL2*-knockdown-induced decrease in 5-FU resistance in HCT15-FR cells. **d** *KLF6* knockdown downregulated the 5-FU-resistance-promoting proteins SEMA3C, β-catenin, TCF1 and c-Myc. This downregulation was impaired by *SEMA3C* overexpression. **e** EdU assays revealed that *SEMA3C* overexpression rescued the decrease in 5-FU resistance in HCT15-FR cells induced by *KLF6* knockdown. **f** Colony formation assays suggested that *SEMA3C* overexpression reversed the KLF6-knockdown-induced decrease in 5-FU resistance in HCT15-FR cells. **g** The 5-FU-resistance-promoting proteins KLF6, H3K23su, SEMA3C and c-Myc were upregulated by TSA (10 µM). This upregulation could be reversed by *KLF6* knockdown. **h** *SEMA3C* knockdown downregulated the expression of the 5-FU-resistance-promoting proteins β-catenin and c-Myc. When β-catenin was overexpressed, this downregulation was impaired. **i** β-Catenin overexpression impaired the SEMA3C-knockdown-induced increase in apoptosis rates in HCT15-FR cells.

**Fig. 9:**
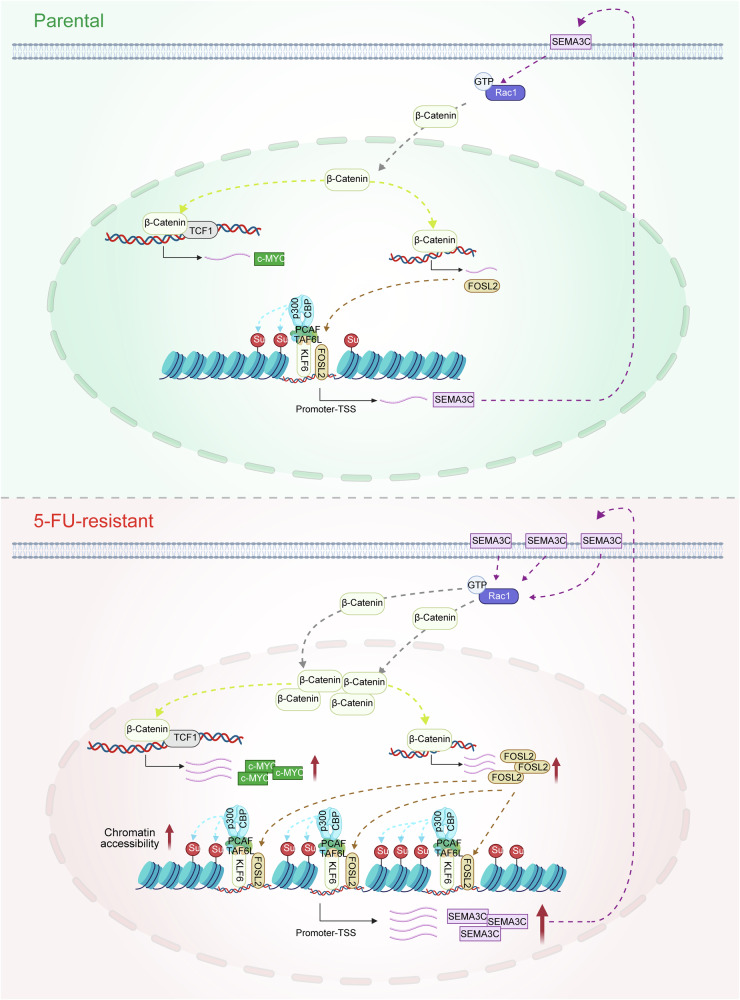
The mechanistic diagram illustrates that KLF6 cooperates with FOSL2 to promote H3K23su levels and enhance 5-FU resistance in HCT15-FR cells. In HCT15-FR cells, the highly expressed KLF6 protein interacts with TAF6L, a component of the PCAF complex. This interaction leads to the recruitment of the PCAF–p300/CBP complex, which, in turn, increases the succinylation of H3K23su. The increased succinylation enhances chromatin accessibility, thereby exposing more promoter regions of the *FOSL2* gene. As a result, the expression of the *FOSL2* target gene *SEMA3C* is upregulated. This upregulation promotes the accumulation of β-catenin in the nucleus by increasing Rac1-GTP levels. Within the nucleus, β-catenin forms a complex with TCF1, leading to the upregulation of c-Myc expression and, consequently, enhancing the 5-FU resistance of colon cancer cells. Moreover, β-catenin also upregulates *FOSL2* expression through a *TCF1*-independent pathway, creating a positive feedback loop that further strengthens the drug resistance of colon cancer cells.

To verify the molecular mechanism underlying SEMA3C-mediated modulation of or *KLF6* functions in 5-FU resistance in HCT15-FR cells, we knocked out *FOSL2* or in HCT15-FR cells. FOSL2 knockdown downregulated the 5-FU-resistance-promoting proteins SEMA3C, β-catenin, TCF1 and c-Myc. When *SEMA3C* was overexpressed, this downregulation was impaired (Fig. 8a and Supplementary Fig. 15c). We then observed that the decrease in 5-FU resistance induced by *FOSL2* knockdown in the nude mouse subcutaneous tumor model could be rescued by *SEMA3C* overexpression. The tumor volume was measured every 4 days after injection (Fig. 8b). Moreover, through colony formation assays, we found that the decrease in viability of HCT15-FR cells induced by *FOSL2* knockdown could be reversed by *SEMA3C* overexpression (Fig. 8c). To confirm how the coagent FOSL2 promotes 5-FU resistance in HCT15-FR cells via SEMA3C, we next knocked out *KLF6* in HCT15-FR cells. We subsequently observed that *KLF6* knockdown downregulated the 5-FU-resistance-promoting proteins SEMA3C, β-catenin, TCF1 and c-Myc. This downregulation was impaired by *SEMA3C* overexpression (Fig. 8d and Supplementary Fig. 15d). The results of the EdU assay revealed that *SEMA3C* overexpression rescued the decrease in viability of HCT15-FR cells induced by *KLF6* knockdown (Fig. 8e). Colony formation assays suggested that *SEMA3C* overexpression reversed the *KLF6*-knockdown-induced decrease in 5-FU resistance in HCT15-FR cells (Fig. 8f). Moreover, a nude mouse subcutaneous tumor model indicated that *SEMA3C* overexpression could rescue the decrease in 5-FU resistance in HCT15-FR cells induced by *KLF6* knockdown. Tumor volumes were measured every 4 days after injection (Supplementary Fig. 9b). We then treated HCT15-FR cells with 0 or 10 µM TSA to increase the expression level of H3K23su. We detected that the 5-FU-resistance-promoting proteins KLF6, FOSL2, H3K23su, SEMA3C and c-Myc were upregulated. This upregulation could be reversed by *KLF6* or *FOSL2* knockdown (Fig. 8g and Supplementary Figs. 9c and 15e). This rescue effect confirmed that *KLF6* mediated the regulatory effect of H3K23su on c-Myc to increase 5-FU resistance in HCT15-FR cells. To verify the molecular mechanism underlying the ability of β-catenin to modulate SEMA3C function in 5-FU resistance in HCT15-FR cells, we knocked down *SEMA3*C in HCT15-FR cells. *SEMA3C* knockdown downregulated the expression of the 5-FU-resistance-promoting proteins β-catenin and c-Myc. When β-catenin was overexpressed, this downregulation was impaired (Fig. 8h and Supplementary Fig. 15f). Furthermore, we found that β-catenin overexpression impaired the SEMA3C-knockdown-mediated increase in apoptosis in HCT15-FR cells (Fig. 8i). Overall, we demonstrated that FOSL2 cooperated with KLF6, which recruited the p300 complex to promote the succinylation of H3K23, which upregulated *SEMA3C* to activate the TCF1-dependent or TCF1-independent Wnt–β-catenin pathway to upregulate *MYC* and *FOSL2*, respectively (Fig. 9).

## Discussion

Lysine succinylation, a recently discovered posttranslational modification of histones, is evolutionarily conserved and is ubiquitous across various species^11^. This epigenetic mark has been implicated in a myriad of cellular regulatory processes, including gene transcription, chromatin structure modulation and metabolic pathway regulation. Notably, specific lysine succinylation sites, such as H3K79su and H3K122su^13,55,56^, have garnered attention; however, the role of lysine succinylation in cancer drug resistance remains largely elusive. In our current study, we investigated the role of genome-wide alterations in H3K23su and its associated DETFs in regulating gene transcription in HCT15-FR colon cancer cells.

Using the CUT&Tag assay^14^, we identified an abundance of high-quality, enriched sites (or peaks) for H3K23su and H3K27ac in both HCT15-Pa and HCT15-FR cells. The majority of these peaks were located within active *cis*-regulatory regions, encompassing promoter TSSs and distal enhancers. This observation is consistent with previous reports suggesting that nucleosome succinylation promotes transcription^12,13,32^, indicating a potential link between the alteration of H3K23su and differential gene transcription in HCT15-FR cells. Comparative analysis between Pa and FR cells led to the identification of DERs for H3K23su and H3K27ac, encompassing GAIN and LOSS regions, characterized by over twofold changes in CUT&Tag signals^16^. A strong positive correlation was revealed through Pearson correlation coefficient analysis between H3K23su DERs and their nearest DEGs. The upregulated DEGs associated with H3K23su GAIN regions were principally associated with pathways related to colorectal cancer, MAPK, Wnt and p53 signaling, whereas the downregulated DEGs associated with H3K23su LOSS regions were notably enriched in the HIF-1 and Hippo signaling pathways. Recent studies have indicated that *IL33*, which is secreted by fibroblasts, promotes the establishment of a proinflammatory microenvironment conducive to breast cancer metastasis to the lungs, a process characterized by a type II inflammatory response^57^. Furthermore, the suppression of *KDM5D* expression confers resistance to docetaxel in prostate cancer cells cultured with dihydrotestosterone^58^. Our findings reveal a substantial overlap between DERs marked by H3K23su and differentially accessible regions identified through ATAC-seq, revealing concurrent trends in their alterations. This convergence suggests a potential synergistic role for H3K23su, H3K27ac and chromatin accessibility in modulating differential gene expression, including the upregulation of *IL33* and the downregulation of *KDM5D*.

TF motif enrichment analysis within H3K23su DERs suggested the potential involvement of FOX, GATA and TEAD family members. By integrating these data with RNA-seq data, we further identified DETF associations with H3K23su changes, including the upregulation of *FOSL2* and *FOXA1* and the downregulation of *HNF1B* and *GRHL2*, which demonstrated increased binding probabilities within the H3K23su GAIN and LOSS regions, respectively. TFs are known to engage in synergistic regulatory mechanisms, exerting their influence on the chromatin state and transcription^59^. A well-documented example of such collaboration occurs when multiple TFs come together to recruit p300/CBP HATs^60^. Our PPI network and coexpression analyses suggest that certain key DETFs associated with H3K23su modifications may collaborate to mediate resistance to 5-FU in HCT15 colorectal cancer cells. To elucidate the mechanism of the identified DETFs, we performed co-IP followed by western blot analysis to detect PPIs. This analysis revealed the absence of an interaction between KLF6 and FOSL2 in HCT15-FR cells. Through IP-MS analysis, we subsequently discovered that TAF6L, a constituent of the PCAF-KAT2B complex, which collaborates with p300 to form the histone acetylase complex, interacts with KLF6. Previous investigations have demonstrated that KLF4 interacts with p300 and recruits the KLF4-p300 complex to the target gene promoter’s KLF4 binding site^61,62^. It is reasonable to hypothesize that KLF6 interacts with TAF6L to recruit PCAF and the p300 acetylase complex. Earlier studies have indicated that a few HATs, including HAT1, KAT2A and CBP/p300, are capable of catalyzing site-specific lysine succinylation of histones^63,64^. Notably, histone succinylation has been implicated in the regulation of transcription and tumor development^64^. Consequently, we used various techniques, including fluorescence confocal microscopy, co-IP and western blot analysis, to investigate the molecular events involved. Our findings revealed that KLF6 recruits PCAF and p300 to increase the succinylation of H3K23, thereby facilitating *FOSL2*-mediated upregulation of *SEMA3C*. Thus, we successfully confirmed the collaborative mechanism between *KLF6* and *FOSL2* in HCT15-FR cells.

Previous studies have demonstrated that *SEMA3C* mediates the activation of the Wnt-β-catenin pathway in glioblastoma^40^. Moreover, *FOSL2* functions as a downstream target gene of the Wnt-β-catenin pathway^46,47^. Based on these findings, we hypothesized that *FOSL2* mediates the upregulation of *SEMA3C*, which, in turn, stimulates the Wnt-β-catenin signaling axis. To substantiate this hypothesis, western blot analysis was used to confirm that *SEMA3C* indeed triggers the activation of the Wnt-β-catenin/TCF1 pathway and enhances the expression of *c-Myc*, thereby fostering resistance to 5-FU in HCT15-FR cells. Furthermore, our data revealed a decrease in *FOSL2* expression after *SEMA3C* knockdown. Upon stimulation with Wnt3a-CM, which induces Wnt/β-catenin activity, we observed the upregulation of *FOSL2*. However, *TCF1* levels remained unaltered despite the increase in *FOSL2* after activation of the Wnt–β-catenin pathway. Based on these observations, we deduced that *SEMA3C* may modulate both TCF1-dependent and TCF1-independent branches of the Wnt–β-catenin pathway, leading to the respective upregulation of *c-Myc* and *FOSL2*.

Subsequently, rescue assays were performed to determine the mechanism by which *SEMA3C* cooperatively affects the interaction between *KLF6* and *FOSL2* and the molecular mechanism underlying *SEMA3C*-mediated modulation of *FOSL2* or *KLF6* functions in 5-FU resistance in HCT15-FR cells.

In conclusion, our study provides deep insights into the importance of histone posttranslational modifications, notably the succinylation of histone H3 at lysine 23, within the context of resistance to 5-FU. Our findings underscore that perturbations in H3K23 succinylation are intimately associated with differential gene expression and can modulate key signaling cascades implicated in oncogenesis. Specifically, the elucidated interplay between *KLF6*, *TAF6L* and *FOSL2*, along with their regulatory influences on SEMA3C and the Wnt-β-catenin pathway, enhancing our understanding of the complex networks underlying cancer drug resistance. These findings emphasize the critical role of histone succinylation in oncology and set the stage for future investigative pursuits and therapeutic developments.

## Supplementary information

Supplementary Information

Supplementary Table 1

Supplementary Table 2

Supplementary Table 3

Supplementary Table 4

Supplementary Table 5

Supplementary Table 6

The top of the list

Statistics of reads mapping

Annotation of CUT&Tag peaks

Annotation for DERs of H3K23su and H3K27ac

Signaling pathways related DEGs positively correlated to H3K23su DERs

Known TF motif enrichment of H3K23su DERs

## Data Availability

All data supporting the findings of this study are available within the Article and its Supplementary Information or from the corresponding author upon reasonable request. All sequencing data have been deposited in the GEO repository under accession number GSE234735.
