# Supplementary material for: KLF6-mediated recruitment of the p300 complex enhances H3K23su and cooperatively upregulates SEMA3C with FOSL2 to drive 5-FU resistance in colon cancer cells
Source: Exp Mol Med. 2025 Mar 13;57(3):667–85. doi: 10.1038/s12276-025-01424-1 (PMC11958781; doi:10.1038/s12276-025-01424-1)
Supplement: Supplementary file 1 — Supplementary Information [file 12276_2025_1424_MOESM1_ESM.pdf]

Supplementary Material

1. Supplementary Figures

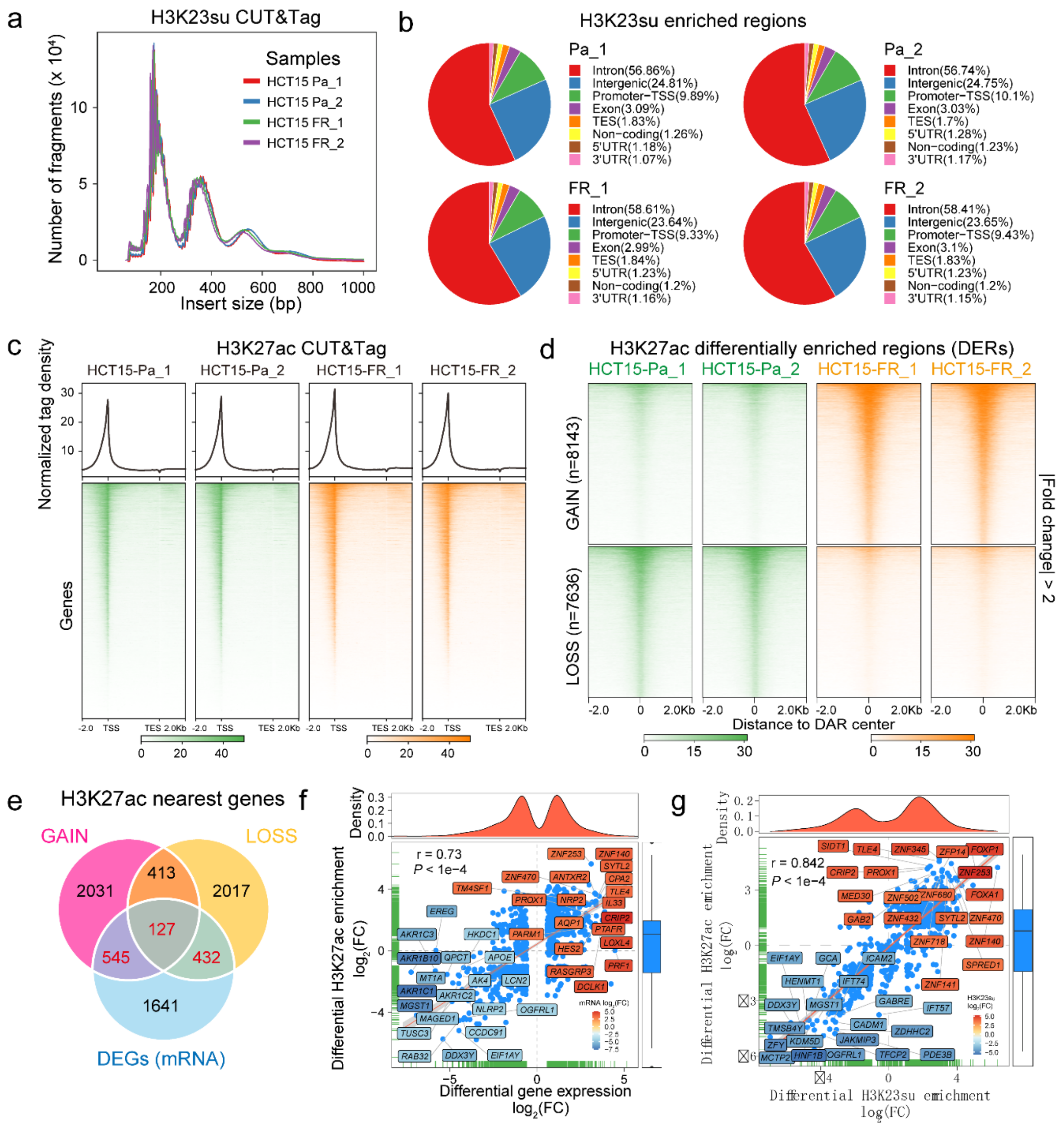

Supplementary Fig. 1. Changes in H3K23su were positively correlated with differential gene expression and H3K27ac enrichment

(a) The insert size distribution shows the H3K23su CUT&Tag fragment length recovered through sequencing.

(b) Pie chart showing the proportion of H3K23su CUT&Tag sites with respect to the indicated genomic regions, including promoter-TSS (define as a 1-kb proximal region centered on the transcription start sites), 5’UTR, 3’UTR, exon, intron, TES (transcription end site) and intergenic regions.

(c) Average profiles (top) and density heatmaps (bottom) indicating H3K27ac CUT&Tag signal across a genomic window of 2 kb upstream of the TSS and 2 kb downstream of the TES.

(d) Heatmap representation of greater (GAIN) and lower (LOSS) H3K27ac CUT&Tag differential differentially enriched regions (DERs) in the HCT15-FR cells versus the parental cells. The upper panel shows the genomic distribution of 8143 H3K27ac GAIN regions; the lower panel shows the distribution of 7636 H3K27ac LOSS regions. signals are displayed in descending order within the 2 kb window from the center of each DER.

(e) Venn diagram illustrating the overlapping genes between the nearest genes of H3K27ac differentially enriched regions (GAIN and LOSS) revealed by CUT&Tag and differentially expressed genes (DEGs) identified by RNA-seq.

(f) Pearson correlation analysis between H3K27ac DERs and their nearest DEGs. Each blue dot represents a nearest DEG. The top- and bottom-ranked 20 DEGs with the most significant difference based on the log2 fold change (FC) are shown in different colors. Pearson correlation coefficient value (r) and *P* value are shown in the figure.

(g) Pearson correlation analysis between H3K27ac and H3K23su related to the same DEG. Each blue dot represents a nearest DEG. The top- and bottom-ranked 20 DEGs with the most significant difference based on the log2 fold change (FC) are shown in different colors. Pearson correlation coefficient value (r) and *P* value are shown in the figure.

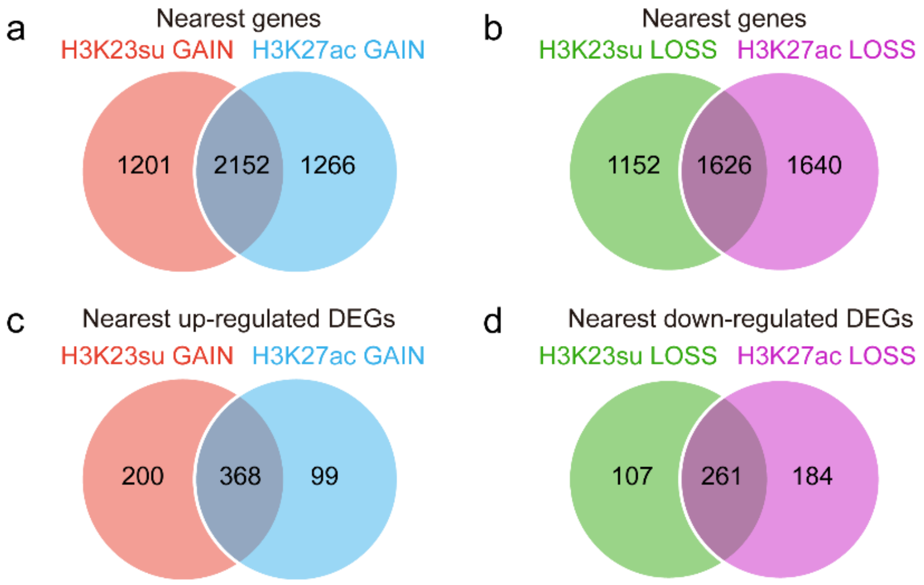

Supplementary Fig. 2. Overlapping genes in proximity to H3K23su and H3K27ac differential enrichment regions (DERs).

(a and b) Overlapping genes of nearest to the GAIN (a) and LOSS (b) H3K23su and H3K27ac DERs.

(c and d) Overlapping up-regulated or down-regulated DEGs nearest to the GAIN (c) or LOSS (d) H3K23su and H3K27ac DERs.

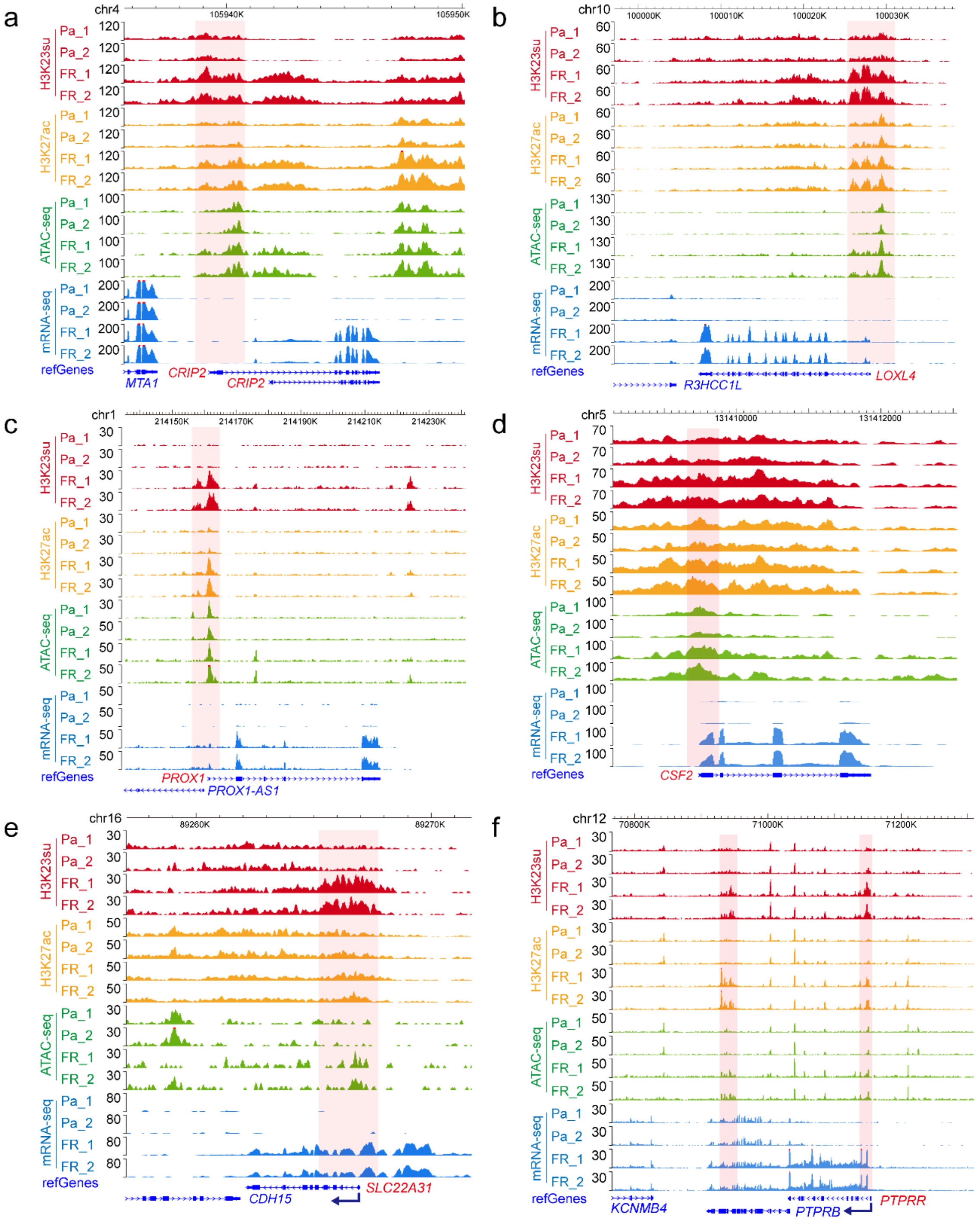

Supplementary Fig. 3. Genomic snapshot of representative up-regulated DEGs associated with H3K23su GAIN regions.

(a-f) The WashU Epigenome Browser tracks show H3K23su (red), H3K27ac (orange), ATAC-seq (green) and RNA-seq (blue) signal of representative up-regulated DEGs, such as *CRIP2*, *LOXL4*, *PROX1* genes. The H3K23su GAIN regions are shaded with red. Dark blue arrows indicate the TSS and the gene transcription direction.

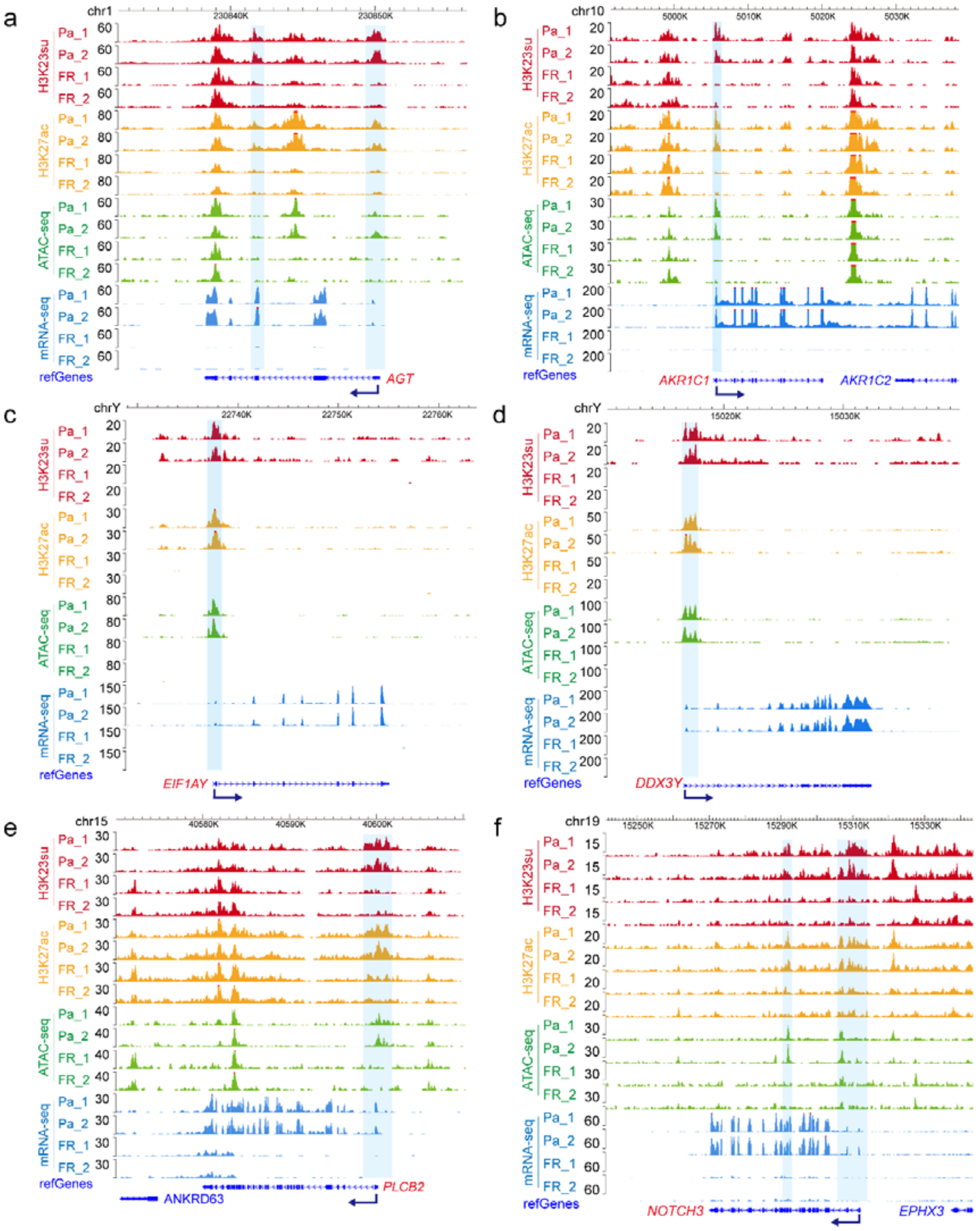

Supplementary Fig. 4. Genomic snapshot of representative down-regulated DEGs associated with H3K23su LOSS regions

(a-f) Genomic snapshots of H3K23su (red), H3K27ac (orange), ATAC-seq (green) and RNA-seq (blue) signal of representative down-regulated DEGs, including *NOTCH3*, *AGT*, *AKR1C1* and *PLCB2* genes. The H3K23su LOSS regions are shaded with blue. Dark blue arrows indicate the TSS and the gene transcription direction.

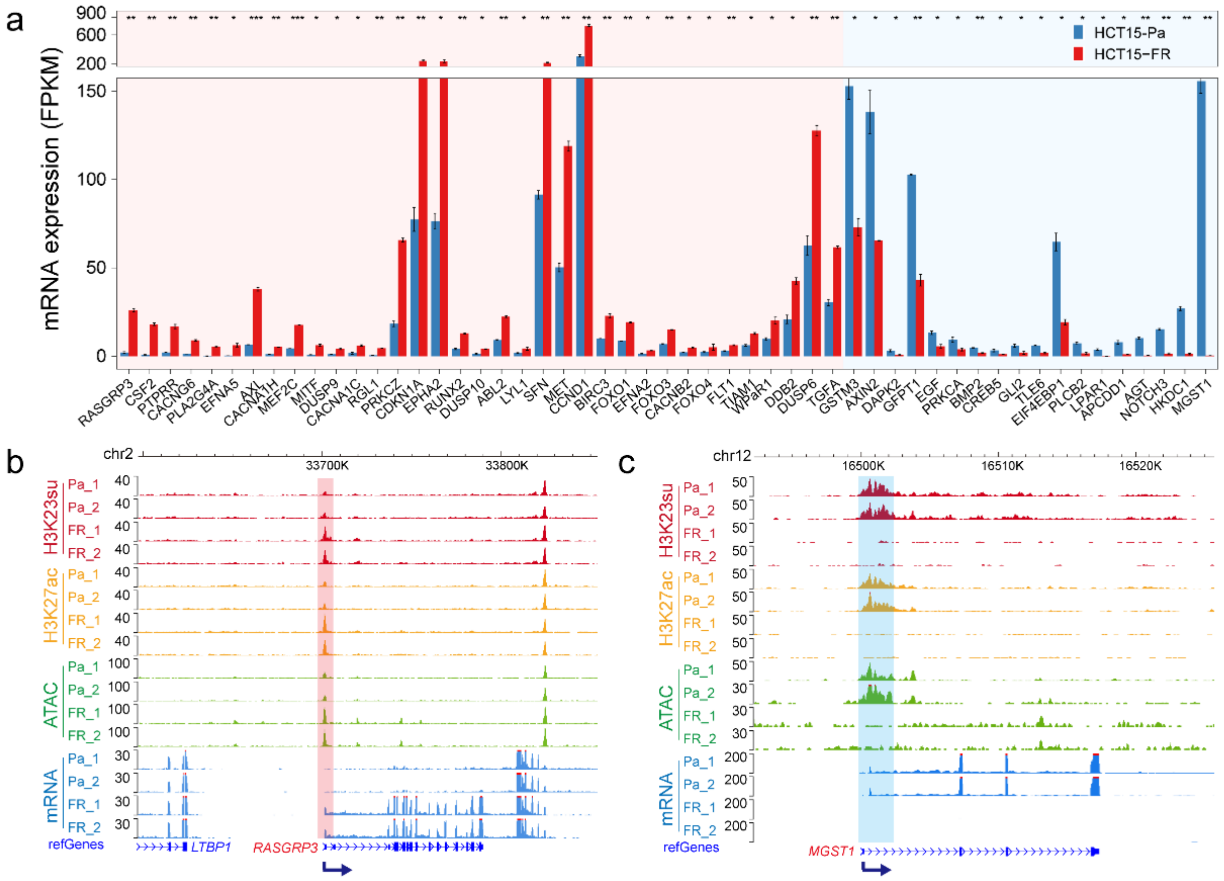

Supplementary Fig. 5. Representative DEGs enriched in top 10 KEGG pathways and positively correlated with H3K23su DERs

(a) Bar plot showing the relative mRNA expression levels of DEGs related to H3K23su GAIN (red shade area) and LOSS (blue shade area) regions which were enriched in top 10 KEGG pathways in Fig. 2 A and 2B. DEGs are listed in descending order based on their log2 fold change values.

(b) The WashU Epigenome Browser tracks show H3K23su (red), H3K27ac (orange), ATAC-seq (green) and mRNA-seq (blue) signal of representative up-regulated *RASGRP3* genes. The H3K23su GAIN regions are shaded with red. Dark blue arrows indicate the TSS and the gene transcription direction.

(c) Genomic snapshots of H3K23su (red), H3K27ac (orange), ATAC-seq (green) and mRNA-seq (blue) signal of representative down-regulated *MGST1* genes. The H3K23su LOSS regions are shaded with blue. Dark blue arrows indicate the TSS and the gene transcription direction.

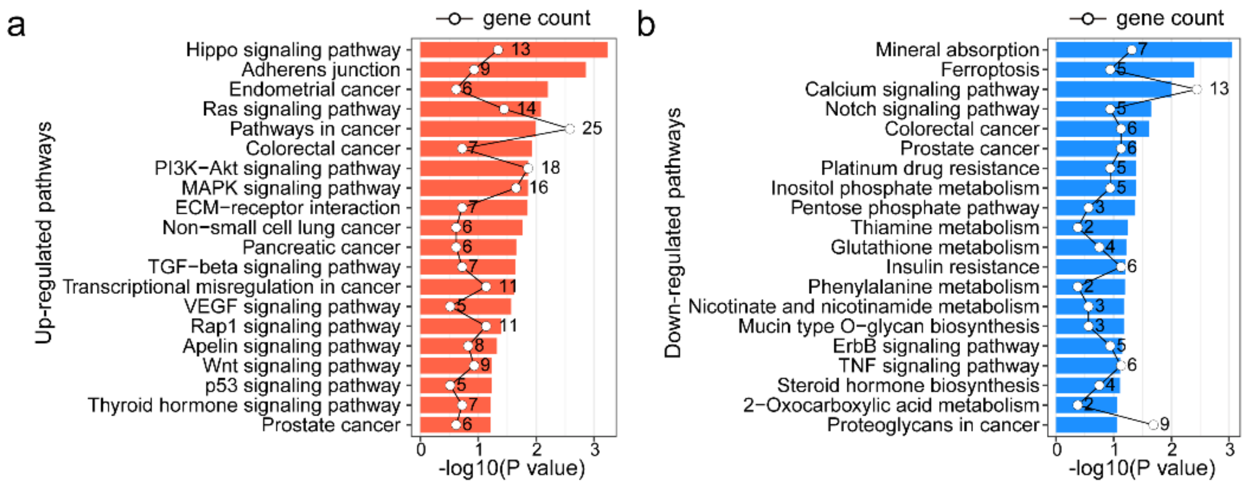

Supplementary Fig. 6. KEGG pathways associated with H3K27ac DERs.

(a and b) Bar plot indicating the top 20 KEGG pathways significantly enriched by up-regulated (a) and down-regulated (b) DEGs nearest to GAIN (a) and LOSS (b) H3K27ac DERs, respectively. Pathways are ordered according to a score corresponding to the -log base 10 of the P value. The polygonal chain in black shows the gene count related to each KEGG pathway.

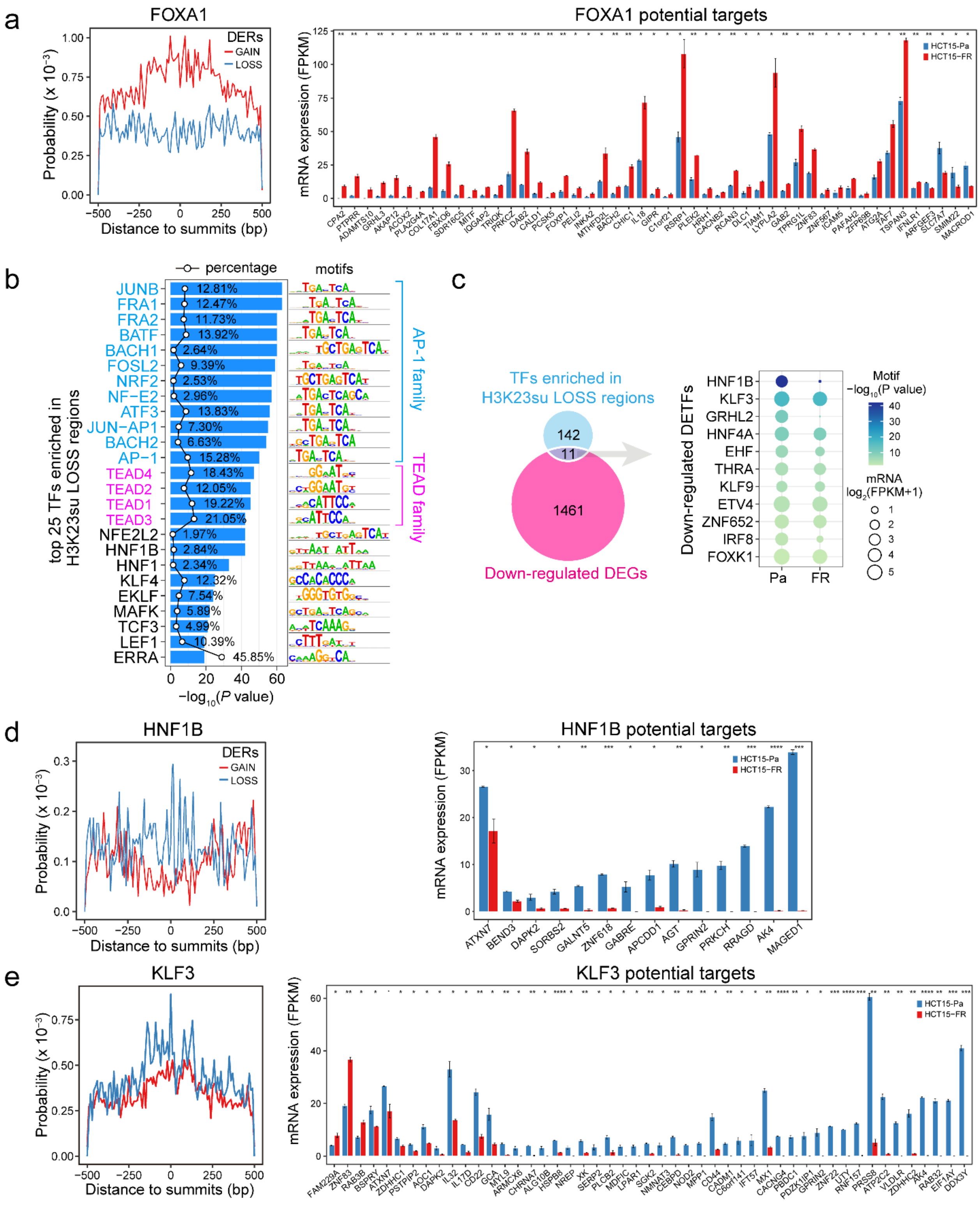

Supplementary Fig. 7. Predicted TFs and their potential target DEGs positively correlated with H3K23su changes

(a) Line charts (left) showing the distribution probability of representative FOXA1 binding motifs around CUT&Tag peak summits in H3K23su DERs. Histograms (right) indicate the expression

levels of FOXA1 target DEGs identified by HOMER (v.4.10). Only DEGs with TFBSs in the promoter-TSS region were included. DEGs are listed in descending order based on log2 fold change.

(b) Top 25 enriched known TF motifs of H3K23su LOSS regions, with *P* values estimated from HOMER (v.4.10). The AP-1, TEAD family members were colored blue and pink, respectively. The percentages of target sequences of DERs with TF motifs are indicated by polygonal chain in black.

(c) Venn diagram indicates the overlap between the corresponding TFs for each motif identified by HOMER (v.4.10) in H3K23su LOSS regions and down-regulated DEGs identified by mRNA-seq. The dot plot (right) shows the identified down-regulated TFs. Dot color represents the P value of TF enrichment, and significant TFs were selected with P value < 0.01. Dot size represents the expression level of the corresponding TFs, log2(FPKM+1).

(d-e) Line charts (left) showing the distribution probability (left) of representative HNF1B and KLF3 binding motifs around CUT&Tag peak summits in H3K23su LOSS regions. Histograms (right) indicate the expression levels of predicted TF (HNF1B and KLF3) target DEGs identified by HOMER (v.4.10) in H3K23su DERs. Only DEGs with TFBSs in the promoter-TSS region were included. DEGs are listed in descending order based on log2 fold change.

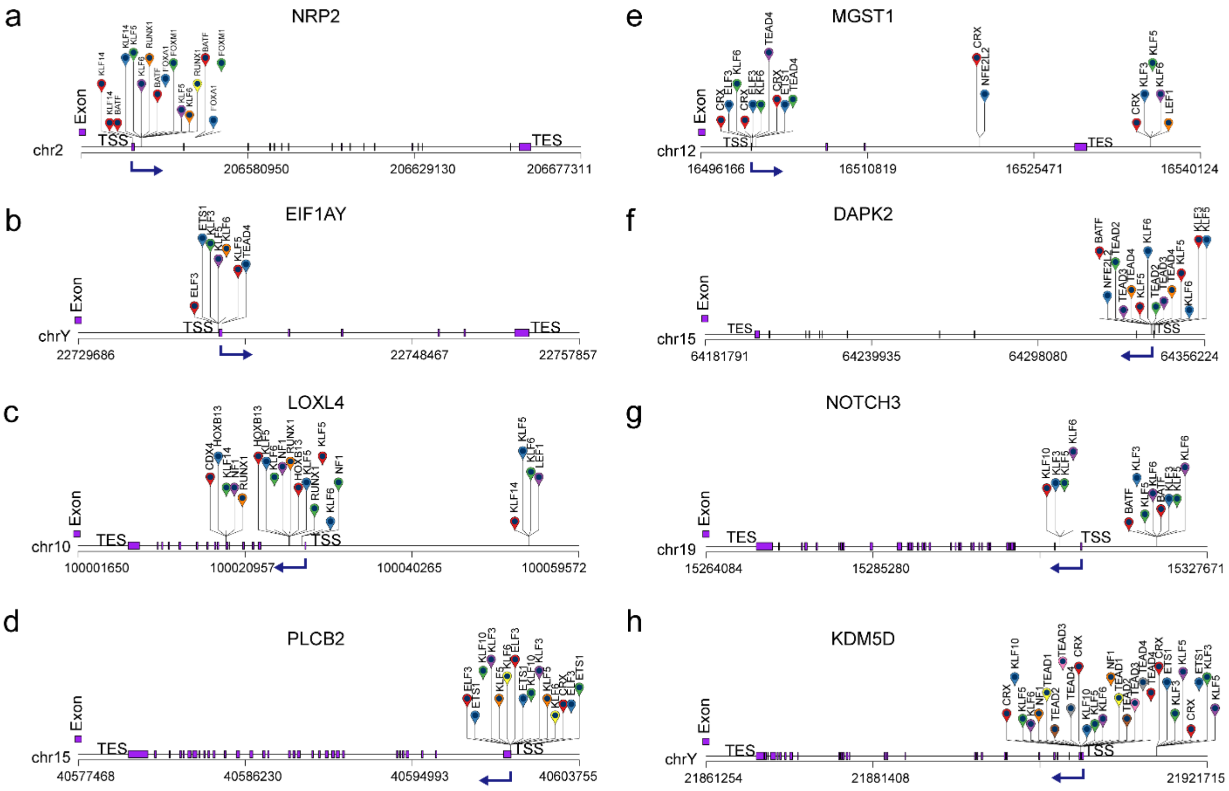

Supplementary Fig. 8. Predicted TF binding sites at representative DEGs

(a-h) Predicted TF binding sites (TFBSs) by HOMER analysis of representative DEGs positively correlated to H3K23su changes. Dark blue arrows indicate the TSS and the gene transcription direction.

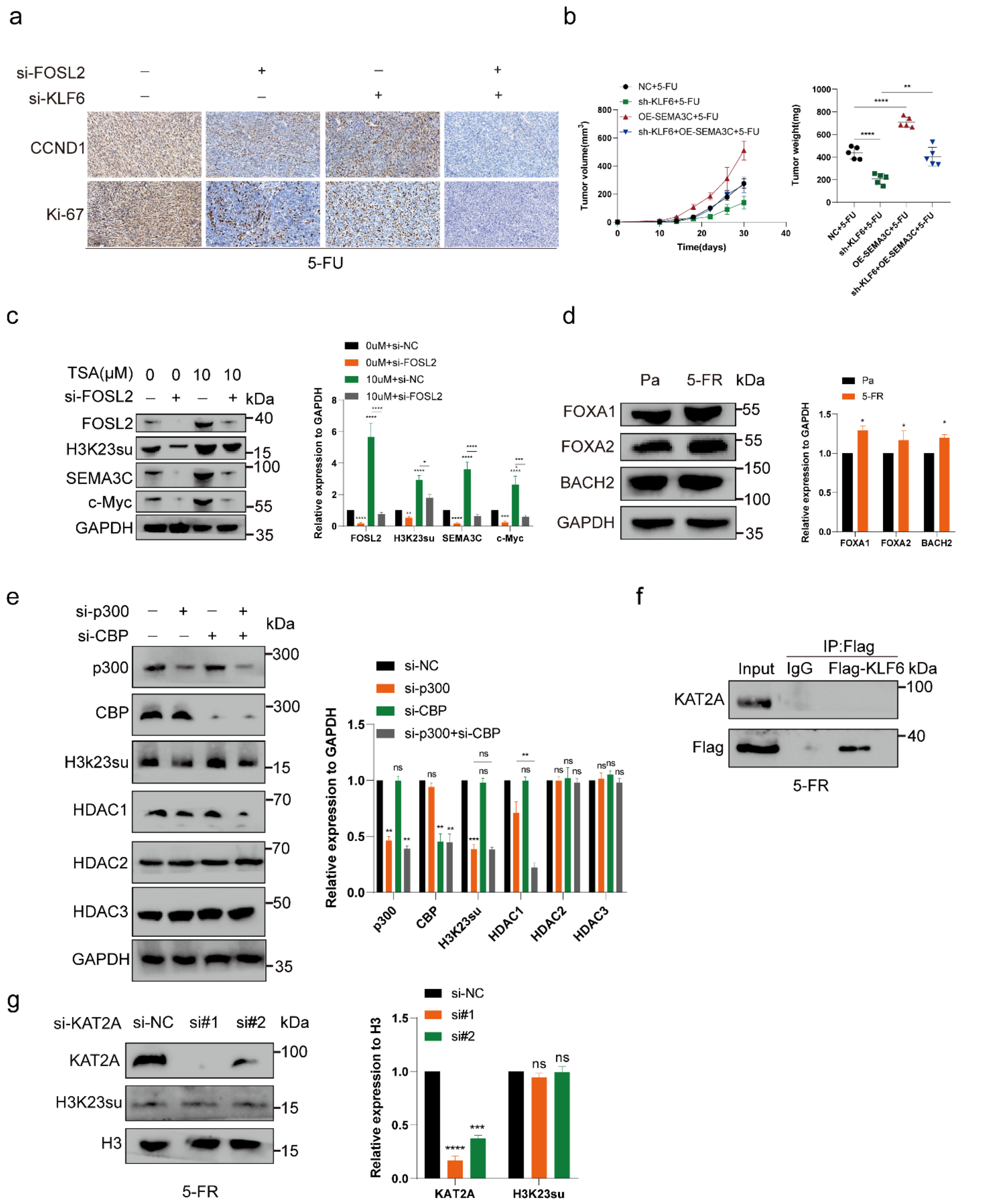

Supplementary Fig. 9. Immunohistochemistry of subcutaneous tumors in nude mice and the expression of FOXA1, FOXA2, and BACH2 in HCT15 Pa and HCT15-FR cells and the effects of CBP and KAT2A on H3K23su

(a) The expression level of Ki-67 and cyclin D1 (CCND1) was significantly downregulated as the drug resistance of colon cancer cells decreased by IHC.

(b) Nude mouse subcutaneous tumor model results indicated that SEMA3C overexpression could rescue the decrease in 5-FU resistance in HCT15-FR cells induced by KLF6 knockdown. The tumor volume was measured every 4 days after injection. The tumor weights of subcutaneous xenografts indicated that SEMA3C overexpression could rescue the decrease in 5-FU resistance in HCT15-FR cells induced by KLF6 knockdown.

(c) HCT15-FR cells were treated with TSA at 0 or 10 μM to enhance the expression level of H3K23su. 5-FU resistance promoting proteins FOSL2, SEMA3C, and c-Myc were upregulated. Above regulation could be reversed by FOSL2 knockdown. 5-FU resistance increase induced by TSA could be reversed by FOSL2 knockdown.

(d) FOXA1, FOXA2 and BACH2 were all up-regulated in HCT15-FR cells compared to HCT15-Pa cells.

(e) H3K23su decreased as p300 knockdown, while H3K23su was not affected as CBP knockdown. HDAC1 was obviously decreased while p300 and CBP were knocked simultaneously. It indicated that HDAC1 was regulated by the redundant effect of p300 and CBP. HDAC2 and HDAC3 were both not affected by p300 or CBP even when p300 and CBP were both knocked.

(f) There was no interaction between KLF6 and KAT2A by Co-IP.

(g) KAT2A knockdown could not affect H3K23su level by WB.

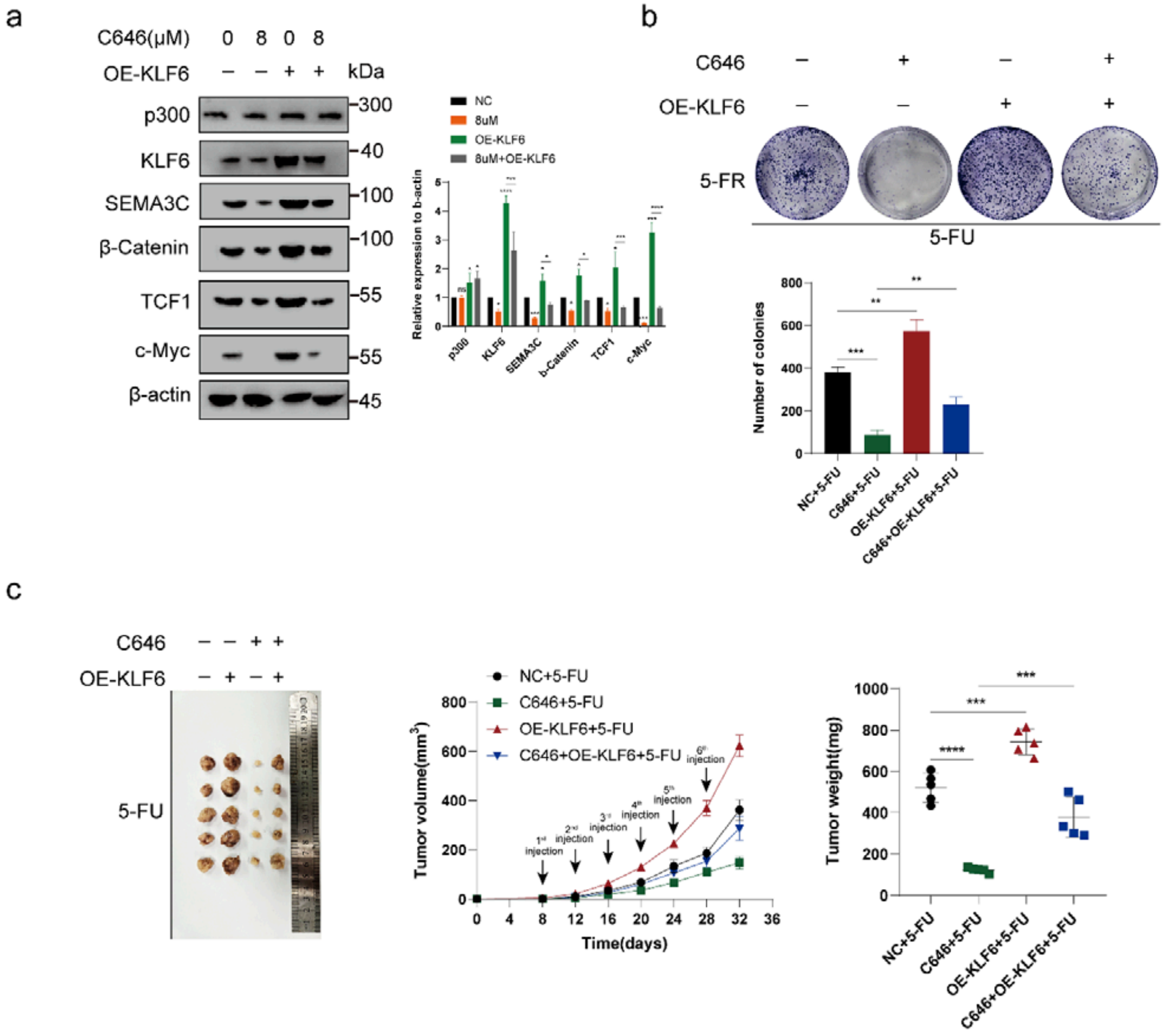

Supplementary Fig. 10. The effect of p300 HAT activity on this signaling using p300 specific inhibitor C646

(a) C646 decreased 5-FU resistance-promoting proteins KLF6, SEMA3C, β-Catenin, TCF1, and c-Myc. This decrease was reversed by KLF6 overexpression.

(b) The colony formation assays suggested that KLF6 overexpression could reverse the C646-induced decrease in 5-FU resistance in HCT15-FR cells.

(c) The nude mouse subcutaneous tumor model indicated that KLF6 overexpression could rescue the decrease in 5-FU resistance in HCT15-FR cells induced by C646. The tumor volume was measured every 4 days after injection. The tumor weights of subcutaneous xenografts. Mice in C646 group and C646 + OE-KLF6 group were intraperitoneally injected with C646 (MCE, 50 nmol/g) every 4 days from the eighth day. C646 was replaced by 10%DMSO+5%Tween80 in NC group and OE-KLF6 group.

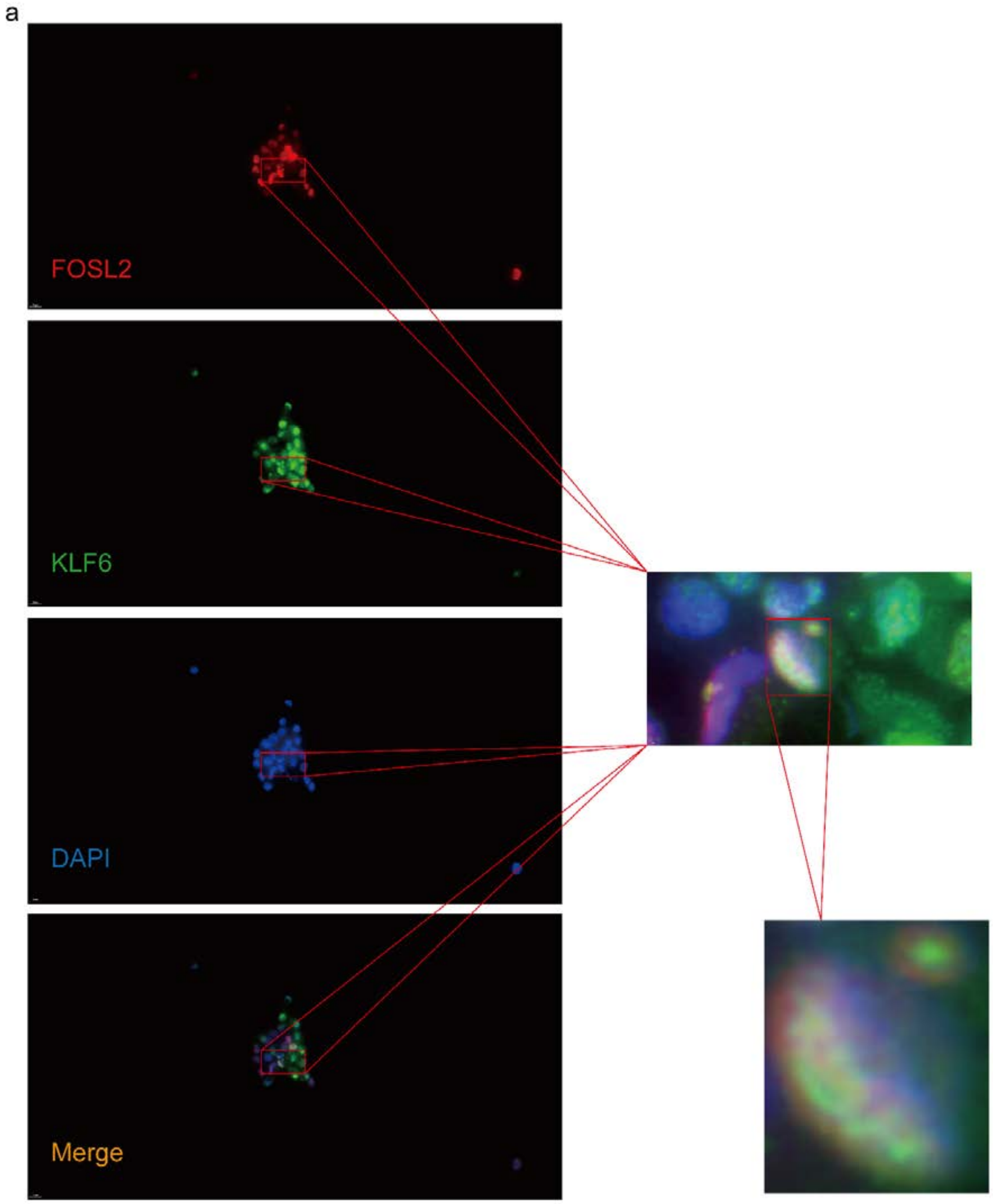

Supplementary Fig. 11. More detailed image details to exhibit the co-localization of FOSL2 and KLF6 in the nucleus of HCT15-FR cells by IF

(a) Co-localization of FOSL2 and KLF6 in the nucleus of HCT15-FR cells by IF.

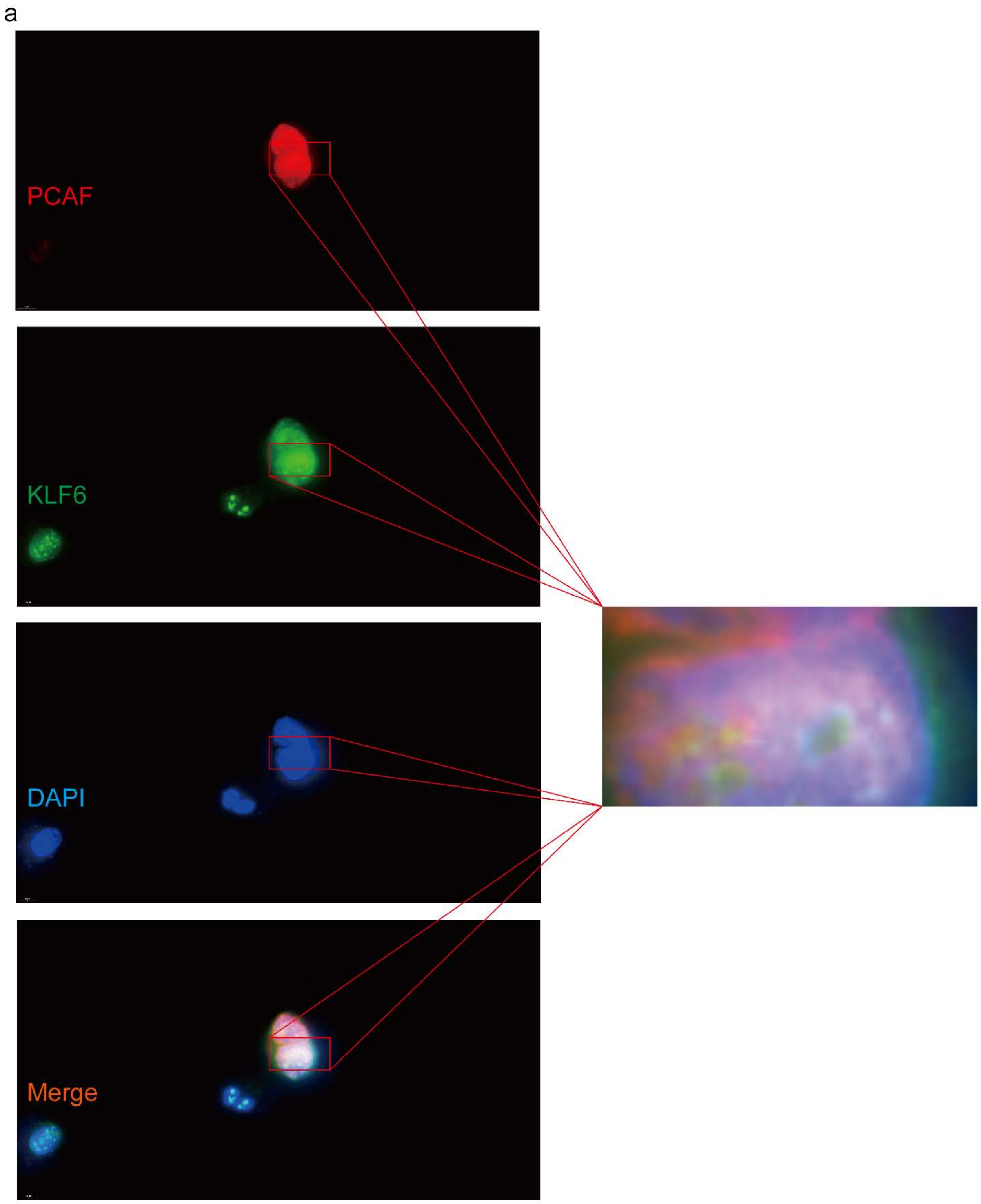

Supplementary Fig. 12. More detailed image details to exhibit the co-localization of PCAF and KLF6 in the nucleus of HCT15-FR cells by IF

(a) Co-localization of KLF6 and PCAF in the nucleus of HCT15-FR cells by IF.

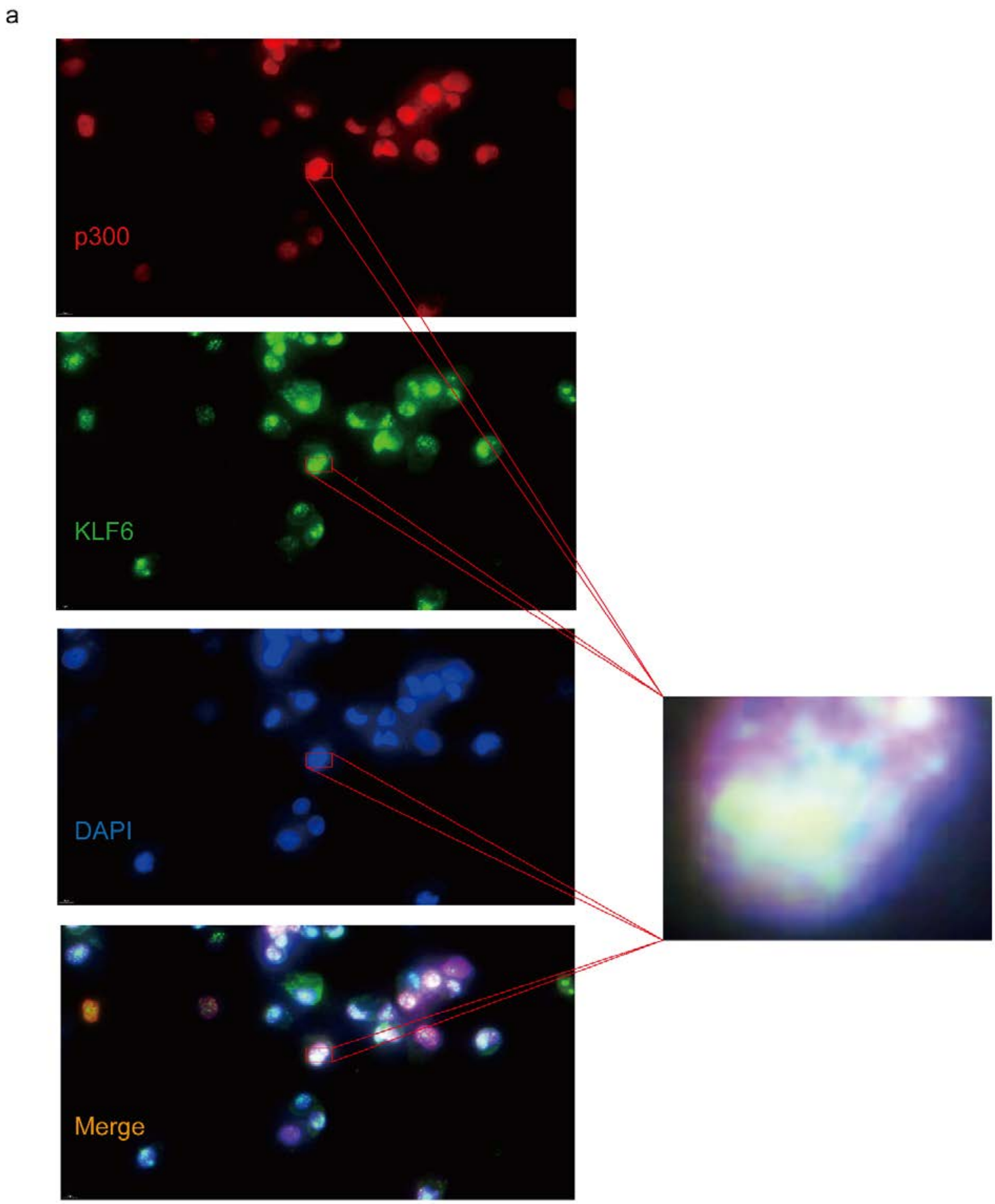

Supplementary Fig. 13. More detailed image details to exhibit the co-localization of p300 and KLF6 in the nucleus of HCT15-FR cells by IF

(a) Co-localization of KLF6 and p300 in the nucleus of HCT15-FR cells by IF.

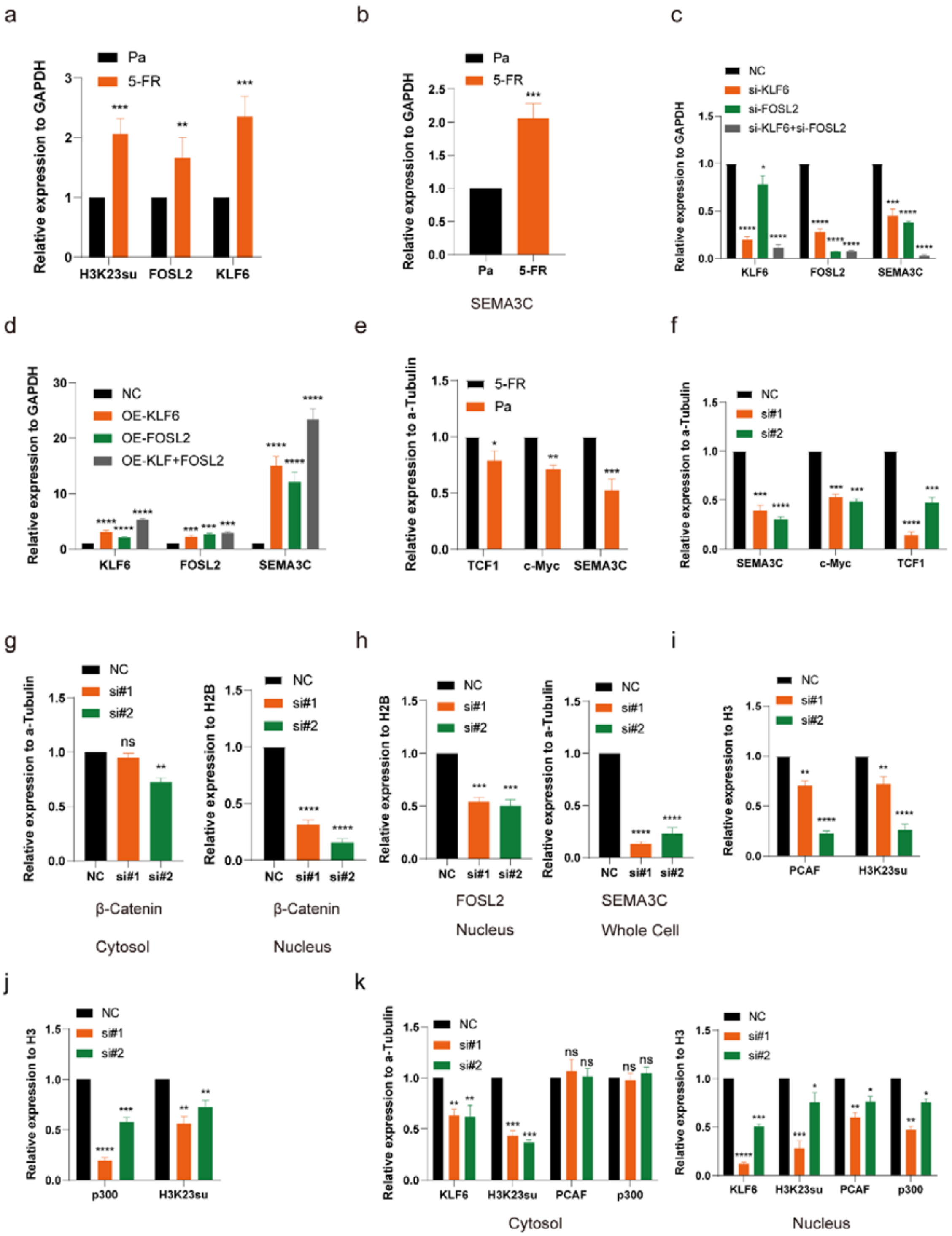

Supplementary Fig. 14. Exhibit WB results through column graph

(a) H3K23su, FOSL2, and KLF6 in HCT15-FR cells were higher than them in HCT15-Pa cells by western blotting.

(b) The expression of SEMA3C was higher in HCT15-FR cells than it in HCT15-Pa cells.

(c) Expression of KLF6, FOSL2, and SEMA3C in NC, FOSL2 knockdown, KLF6 knockdown, and FOSL2 and KLF6 knockdown group in HCT15-FR cells. SEMA3C declined as FOSL2 or KLF6 knockdown. SEMA3C declined more obviously as FOSL2 and KLF6 were knocked together.

(d) Expression of FOSL2, KLF6, and SEMA3C in NC, FOSL2 overexpression, KLF6 overexpression, and FOSL2 and KLF6 overexpression group in HCT15-Pa cells. SEMA3C increased as FOSL2 or KLF6 overexpression and SEMA3C increased more obviously as FOSL2 and KLF6 overexpression together in HCT15-Pa cells.

(e) TCF1 and c-Myc were up-regulated in HCT15-FR cells by WB.

(f) TCF1 and c-Myc decreased as SEMA3C knockdown in HCT15-FR cells by WB.

(g) β-catenin obviously decreased in nucleus as SEMA3C knockdown in HCT15-FR cells by WB.

(h) FOSL2 reduced in nucleus of HCT15-FR cells with SEMA3C knockdown.

(i) H3K23su decreased as PCAF knockdown by WB.

(j) H3K23su decreased as p300 knockdown by WB.

(k) H3K23su, PCAF, and p300 decreased in the nucleus of HCT15-FR cells with KLF6 knockdown, while PCAF and p300 remained unchanged in cytosol.

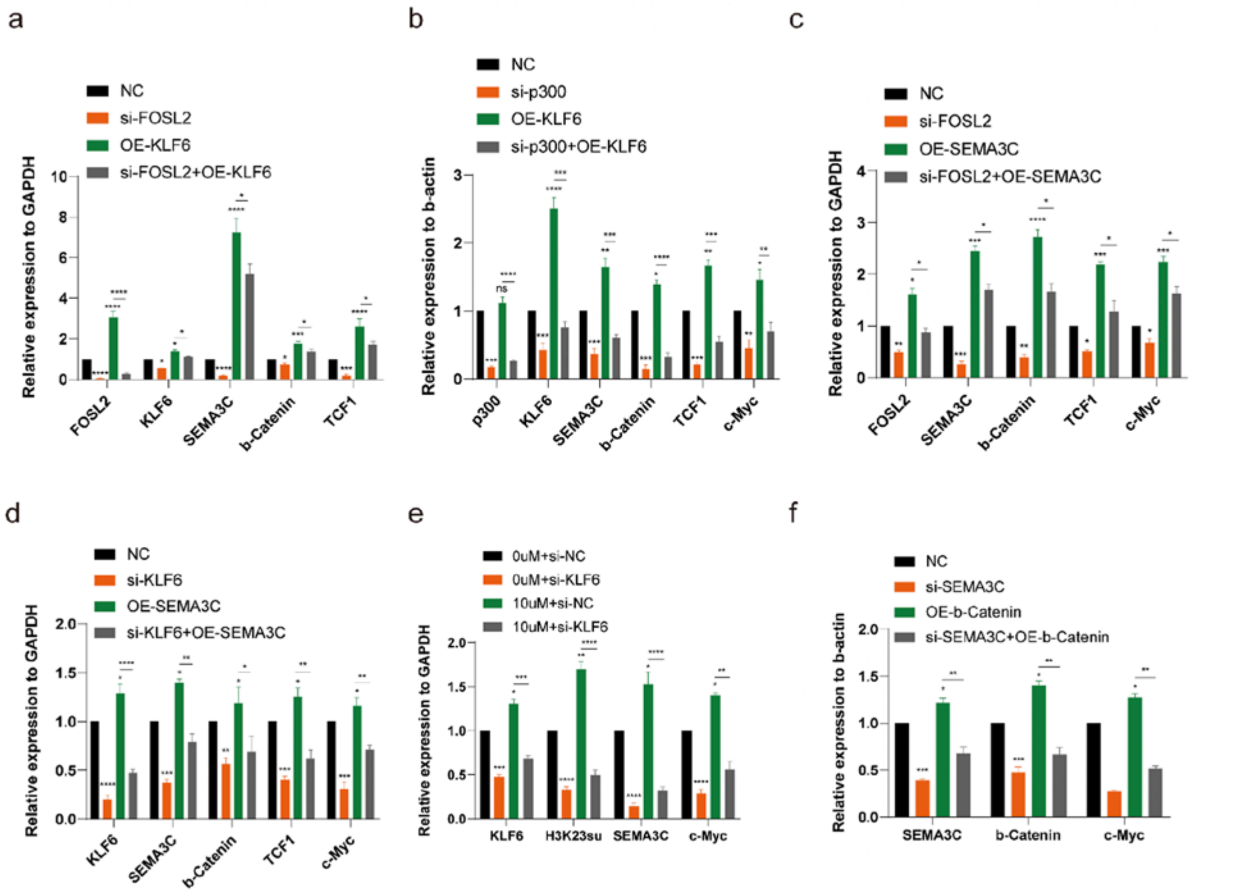

Supplementary Fig. 15. Exhibit WB results through column graph

(a) FOSL2 knockdown in HCT15-FR cells downregulated 5-FU resistance promoting proteins SEMA3C, β-Catenin, TCF1, and c-Myc. When KLF6 was overexpressed, this downregulation was impaired.

(b) SEMA3C, β-Catenin, TCF1, and c-Myc, which induced the increase in 5-FU resistance of HCT15-FR cells were down-regulated by si-p300. This downregulation was reversed by KLF6 overexpression.

(c) FOSL2 knockdown downregulated 5-FU resistance promoting proteins SEMA3C, β-Catenin, TCF1, and c-Myc. When SEMA3C was overexpressed, this downregulation was impaired.

(d) KLF6 knockdown downregulated 5-FU resistance promoting proteins SEMA3C, β-Catenin, TCF1, and c-Myc. And this downregulation was impaired by SEMA3C overexpression.

(e) 5-FU resistance promoting proteins KLF6, H3K23su, SEMA3C, and c-Myc were upregulated by TSA (10uM). And this upregulation could be reversed by KLF6 knockdown.

(f) SEMA3C knockdown downregulated 5-FU resistance promoting proteins β-Catenin and c-Myc. When β-Catenin was overexpressed, this downregulation was impaired.

2. Supplementary Tables

Supplementary Tables 1 - 6 will be provided in Excel format due to the extensive volume of data.

Supplementary Table 7. siRNA sequences used in this study.

siRNA name	Sequence
si-FOSL2 sense	5′-GCGCTCTGTCATCAAGCCCAT-3′<br>5′-CAGCAGAAATTCCGGGTAGAT-3′
si-KLF6 sense	5′-ACTCAGATGTCAGCAGCGAAT-3′<br>5′-CAGGAAGATCTGTGGACCAAA-3′
si-SEMA3C sense	5′-GCCAAGATCAACTTCAAAGTT-3′<br>5′-CGTGTAATTCAGACTTTCAAT-3′
si-PCAF sense	5′-GCAGACTTACAGCGAGTCTTT-3′<br>5′-CGAACTCTAATCCTCACTCAT-3′
si-p300 sense	5′-GCCTTCACAATTCCGAGACAT-3′<br>5′-GCCAGCCTCAAACTACAATAAA-3′
siRNA-NC sense	5′-UUCUCCGAACGUGUCACGUTT-3′
si-CBP sense	5′-AACAGGCAGCCAGCACCTCTG-3′<br>5′-GATGCTGCTTCCAAACATA-3′
si-KAT2A	5′-CACATCATCAAGAAGCAGAAA-3′<br>5′-CCTGGAGAAGTTCTTCTACTT-3′

Supplementary Table 8. Antibodies used for western blotting.

Antibody	Company	Cat. No.	Species	Dilution
H3K23su	PTM BIO	PTM-422	Mouse	1:1000
Gapdh	Cell signaling	1.1.1.12118S	Rabbit	1:1000
α-tubulin	Cell signaling	1.1.1.22144S	Rabbit	1:1000
FOSL2	Proteintech	15832-1-AP	Rabbit	1:5000
KLF6	Proteintech	67297-1-Ig	Mouse	1:1000
SEMA3C	Proteintech	19242-1-AP	Rabbit	1:1000
c-Myc	Cell signaling	18583	Rabbit	1:1000
TCF1	Cell signaling	2203S	Rabbit	1:1000
β-catenin	Cell signaling	8480S	Rabbit	1:1000
FLAG-Tag	Proteintech	20543-1-AP	Rabbit	1:2000
Rac1	Cytoskeleton	Cat#ARC03	Rabbit	1:1000

H2B	Cell signaling	12364S	Rabbit	1:2000
TAF6L	Proteintech	67569-1-Ig	Mouse	1:2000
P300	Proteintech	20695-1-AP	Rabbit	1:1000
PCAF	Proteintech	28770-1-AP	Rabbit	1:1000
FOXA1	Proteintech	20411-1-AP	Mouse	1:3000
FOXA2	Proteintech	22474-1-AP	Rabbit	1:2000
BACH2	Proteintech	27635-1-AP	Rabbit	1:2000
HDAC1	Cell signaling	5356T	Mouse	1:1000
HDAC2	Cell signaling	57156T	Rabbit	1:1000
HDAC3	Cell signaling	85057T	Rabbit	1:1000
CBP	Cell signaling	7389T	Rabbit	1:1000
KAT2A	Cell signaling	3305T	Rabbit	1:1000

Supplementary Table 9. ChIP qPCR Primer sequences used in this study.

**Primer name**	**Sequence**
RAMP1 forward	5′-ACCCAGTTCCAGGTAGACAT-3′
RAMP1 reverse	5′- CAGCTTCTCCGCCATGTG-3′
MX2 forward	5′-AATTGACTTCTCCTCCGGTA-3′
MX2 reverse	5′- TACCGGAGGAGAAGTCAATT-3′
SEMA3C forward	5′- TTTGCGTGTTGGTTGGAGTAT -3′
SEMA3C reverse	5′- TCCTGTAGTCTAAAGGATGGTGG -3′
TFF3 forward	5′- CCCTGCAGGAAGCAGAAT-3′
TFF3 reverse	5′-GGGAGCAAAGGGACAGAA A-3′
OBSL1 forward	5′-AGGGCGGTGTTGGAGGTGACTGT-3′
OBSL1 reverse	5′-TGGATGACTAGGCTGTGGGTGGTG-3′
